# Microbiomes of *Thalassia testudinum* throughout the Atlantic Ocean, Caribbean Sea, and Gulf of Mexico are influenced by site and region while maintaining a core microbiome

**DOI:** 10.3389/fmicb.2024.1357797

**Published:** 2024-02-23

**Authors:** Kelly Ugarelli, Justin E. Campbell, O. Kennedy Rhoades, Calvin J. Munson, Andrew H. Altieri, James G. Douglass, Kenneth L. Heck, Valerie J. Paul, Savanna C. Barry, Lindsey Christ, James W. Fourqurean, Thomas K. Frazer, Samantha T. Linhardt, Charles W. Martin, Ashley M. McDonald, Vivienne A. Main, Sarah A. Manuel, Candela Marco-Méndez, Laura K. Reynolds, Alex Rodriguez, Lucia M. Rodriguez Bravo, Yvonne Sawall, Khalil Smith, William L. Wied, Chang Jae Choi, Ulrich Stingl

**Affiliations:** ^1^Department of Microbiology and Cell Science, Ft. Lauderdale Research and Education Center, University of Florida, Davie, FL, United States; ^2^Department of Biological Sciences, Institute of Environment, Coastlines and Oceans Division, Florida International University, Miami, FL, United States; ^3^Smithsonian Marine Station, Fort Pierce, FL, United States; ^4^Institute for the Oceans and Fisheries, University of British Columbia, Vancouver, BC, Canada; ^5^Department of Ecology and Evolutionary Biology, University of California, Santa Cruz, Santa Cruz, CA, United States; ^6^Department of Environmental Engineering Sciences, University of Florida, Gainesville, FL, United States; ^7^Smithsonian Tropical Research Institute, Panama City, Panama; ^8^The Water School, Florida Gulf Coast University, Fort Myers, FL, United States; ^9^Dauphin Island Sea Lab, University of South Alabama, Dauphin Island, AL, United States; ^10^University of Florida, Institute of Food and Agricultural Sciences Nature Coast Biological Station, University of Florida, Cedar Key, FL, United States; ^11^International Field Studies, Inc., Andros, Bahamas; ^12^College of Marine Science, University of South Florida, St. Petersburg, FL, United States; ^13^Soil and Water Sciences Department, University of Florida, Gainesville, FL, United States; ^14^Department of Environment and Natural Resources, Government of Bermuda, Hamilton Parish, Bermuda; ^15^Center for Advanced Studies of Blanes (Spanish National Research Council), Girona, Spain; ^16^Soil, Water and Ecosystem Sciences Department, University of Florida, Gainesville, FL, United States; ^17^King Abdullah University of Science and Technology, Thuwal, Saudi Arabia; ^18^Bermuda Institute of Ocean Sciences (BIOS), St. George's, Bermuda

**Keywords:** *Thalassia*, seagrass microbiome, amplicon sequencing, Caribbean, seagrass beds, seagrass, core microbiome

## Abstract

Plant microbiomes are known to serve several important functions for their host, and it is therefore important to understand their composition as well as the factors that may influence these microbial communities. The microbiome of *Thalassia testudinum* has only recently been explored, and studies to-date have primarily focused on characterizing the microbiome of plants in a single region. Here, we present the first characterization of the composition of the microbial communities of *T. testudinum* across a wide geographical range spanning three distinct regions with varying physicochemical conditions. We collected samples of leaves, roots, sediment, and water from six sites throughout the Atlantic Ocean, Caribbean Sea, and the Gulf of Mexico. We then analyzed these samples using 16S rRNA amplicon sequencing. We found that site and region can influence the microbial communities of *T. testudinum*, while maintaining a plant-associated core microbiome. A comprehensive comparison of available microbial community data from *T. testudinum* studies determined a core microbiome composed of 14 ASVs that consisted mostly of the family Rhodobacteraceae. The most abundant genera in the microbial communities included organisms with possible plant-beneficial functions, like plant-growth promoting taxa, disease suppressing taxa, and nitrogen fixers.

## Introduction

Seagrass meadows form ecologically important ecosystems that are at risk due to environmental change (Waycott et al., 2009). They provide food and habitat for marine animals, along with other benefits such as water quality improvement and carbon sequestration (reviewed in Dewsbury et al., 2016). However, many meadows are threatened by various environmental stressors, such as hypersalinity, hypoxia, high temperatures, eutrophication, and disease (Koch et al., 2007a; Barry et al., 2017; Bishop et al., 2017; Ugarelli et al., 2017). Nevertheless, some species exhibit a surprising capacity for resilience (Unsworth et al., 2015). For example, *Thalassia testudinum*, or turtlegrass, one of the most prominent seagrasses in the Caribbean, shows a remarkable ability to adapt to varying sediment conditions and levels of sediment anoxia (Koch et al., 2007b). Furthermore, *T. testudinum* can also adapt to prolonged nutrient stress by changing their metabolism and lipid profile (Koelmel et al., 2019), as well as their photosynthetic efficiency and biomass partitioning (Fourqurean et al., 1992; Lee and Dunton, 2000; Barry et al., 2017).

Distinct microbial communities live on and within the leaves, rhizomes, and roots of seagrasses, and provide distinct benefits to the plant (reviewed in Ugarelli et al., 2017), possibly contributing to physiological stress responses and overall resilience. Oftentimes, stressors that affect seagrasses also affect their microbiome. In *Thalassia* spp., increasing inorganic nitrogen (N) can lead to changes in the microbial communities of the rhizosphere (Zhou et al., 2021), while changes in temperature, light (Vogel et al., 2021b), water depth, and salinity (Vogel et al., 2020) can affect the phyllosphere communities. Microbial communities vary among leaf, root, sediment, and water samples (Cúcio et al., 2016; Fahimipour et al., 2017; Rotini et al., 2017; Crump et al., 2018; Hurtado-McCormick et al., 2019; Banister et al., 2021), potentially due to the distinct micro-environments and the biogeochemical processes occurring at each. For instance, the leaves release dissolved organic carbon and oxygen (Wetzel and Penhale, 1979; Borum et al., 2006) along with other exudates that may select for different microbes compared to seawater communities. The root system also releases dissolved organic carbon and oxygen (Wetzel and Penhale, 1979; Borum et al., 2006) which is especially “selective” in anoxic sediments and alters the redox conditions which influences the microbial community composition (reviewed in Ugarelli et al., 2017). Some of the most common taxa present in the microbiome of seagrasses include nitrogen fixers (reviewed in Ugarelli et al., 2017), sulfate-reducers (some of which can fix nitrogen; Küsel et al., 1999), and sulfide-oxidizers (Ettinger et al., 2017; Martin et al., 2019), all of which can be considered part of a core microbiome and provide benefits to the seagrass host.

A core microbiome can be defined as microbial taxa that are present across multiple samples of the same host species. The way core microbiomes are defined differs across studies, depending on the requirements for inclusion of taxa (i.e., both presence and abundance vs. only presence; Shade and Handelsman, 2012). In some cases, researchers consider the core microbiome to be functional rather than taxonomic, meaning that functional roles can be fulfilled by taxa of different species (e.g., any N-fixer could be considered part of the core microbiome, as long as N fixation is found in all samples of the host species reviewed in Lemanceau et al., 2017; Jones et al., 2019; Neu et al., 2021). In several seagrass studies, despite the differences in microbial community compositions at different locations, indications of core microbiomes exist. The core microbiome in these studies is generally defined as microbes that are present in most samples and is usually classified to family level (Cúcio et al., 2016; Roth-Schulze et al., 2016; Bengtsson et al., 2017; Hurtado-McCormick et al., 2019; Banister et al., 2021; Rotini et al., 2023).

Studies show that the leaf communities resemble the water communities for certain seagrass species, like *Zostera marina* (Fahimipour et al., 2017) and *Halophila stipulacea* (Conte et al., 2021b); however, this is not generally the case in other species like *Halophila ovalis* and *Posidonia australis* (Roth-Schulze et al., 2016), nor *T. testudinum* (Ugarelli et al., 2019; Vogel et al., 2020), where the leaf communities differ distinctly from the water communities. Other species of seagrasses, like *Posidonia oceanica*, have been shown to both have distinct leaf microbial communities from the water communities (Kohn et al., 2020), or similar leaf and water communities (Conte et al., 2021b) depending on the study. Seagrasses are a polyphyletic group of plants (Les et al., 1997), and the differing physiology of each species may be in part responsible for the composition of their microbiomes (Conte et al., 2021b). Not only do microbial communities differ among sample types, but also among sites (Mvungi and Mamboya, 2012; Cúcio et al., 2016; Bengtsson et al., 2017; Banister et al., 2021) that differ in environmental conditions. Moreover, microbial communities have also been shown to vary temporally based on seasons (Korlević et al., 2021) and even time of day (Rotini et al., 2020, reviewed in Conte et al., 2021a). Water temperature, depth, salinity, and phosphate concentrations have all been shown to influence the microbial communities of *T. testudinum* (Vogel et al., 2020, 2021a). Other environmental factors such as pH (Hassenrück et al., 2015; Banister et al., 2021) and nutrient enhancement via fertilization influence the microbiome of other seagrass species (Wang L. et al., 2020), but these relationships are poorly understood for *T. testudinum*. Furthermore, no comparisons have been made of the microbiome of *T. testudinum* across broader spatial scales (within or across regions, which here we define as distinct bodies of water). Given the breadth of environmental variability of marine water bodies at larger geographic scales, these studies would enhance our understanding of the core microbiome of this seagrass species as well its influence on the physiological responses of *T. testudinum* to environmental conditions. There is only one large scale study available on the seagrass microbiome and it focuses on the leaves, roots, sediment, and water samples of *Z. marina* meadows throughout the world (Fahimipour et al., 2017). They found that the microbiome differs by site and sample type, and they found evidence of a core microbiome, at least in the roots. No such study is available for other seagrass species.

**Figure 1:**
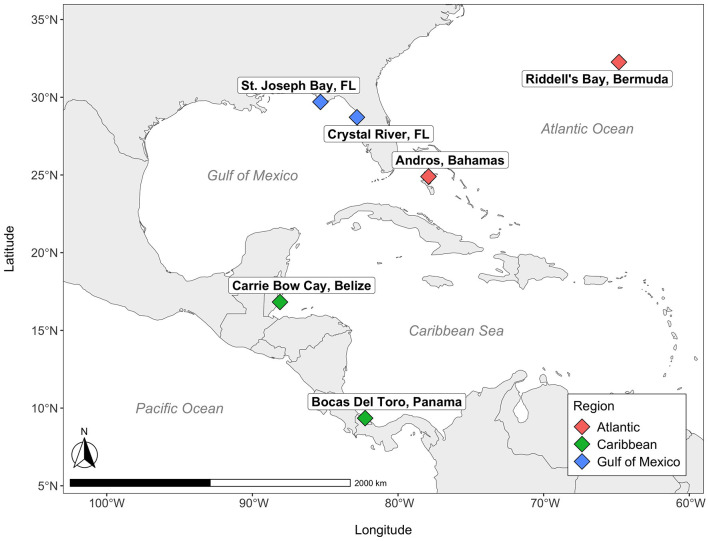
Map of seagrass sites. Colors correspond to various regions across the Western Atlantic.

Here, we investigate the compositional microbiome of *T. testudinum* at six sites across three regions that cover a large portion of its geographic range (Phillips and Meñez, 1988): Andros (Bahamas) and Riddell's Bay (Bermuda) in the Atlantic Ocean; Carrie Bow Cay (Belize) and Bocas del Toro (Panama) in the Caribbean Sea; and two sites in Florida, USA (Crystal River and St. Joseph Bay) in the Gulf of Mexico (Figure 1). The objective of this study was to determine whether the microbial communities of *T. testudinum* across a large geographical range differ by site and region, and through comparisons with other studies, to determine if *T. testudinum* exhibits evidence of a leaf core microbiome and what organisms it comprises.

## Methods

### Site description and sampling methods

Samples of water, sediment, leaves and roots were collected, in that order, from a single seagrass meadow at each of six distinct sites distributed across the Gulf of Mexico and Greater Caribbean: Andros (Bahamas); Carrie Bow Cay (Belize); Riddell's Bay (Bermuda); Crystal River (FL USA); Bocas del Toro (Panama); St. Joseph Bay (FL, USA; Figure 1). These sites were a subset of a larger seagrass network (Campbell et al., 2024), and were collected between the late summer and early fall of 2018. The meadow within each site was selected by adhering to a standardized set of criteria: (1) depth (< 3 m); (2) plant community composition (turtlegrass, >50% relative abundance); (3) meadow dimensions (minimum 25 m × 25 m); (4) low wave energy/storm exposure. These sites also represent a range of different sediment types: Andros (Bahamas), Carrie Bow Cay (Belize), and Riddell's Bay (Bermuda) all have high-carbonate sediments, Crystal River (FL, USA) has intermediate-carbonate sediment, Bocas del Toro (Panama) contains mixed carbonate-siliciclastic sediment, and St. Joseph Bay (FL, USA) has siliciclastic, or low-carbonate sediment (as identified in Fourqurean et al., 2023). Each site contained a grid of 50 experimental seagrass plots (each 0.25 m^2^) dominated by *T. testudinum*. From these 50 plots, we sampled 10 plots at each site. Five were unmanipulated controls, while the other five received fertilizer amendments via the addition of 300 g of Osmocote (NPK 14-14-14). One of our original goals was to examine the effects of nutrient enrichment on the seagrass microbiome; however, the effectiveness of enrichment (particularly for N) was variable on the plants across our specific subset of sites from the network, and consequently, nutrient treatment did not exhibit any detectable effects on the microbial community composition, except for the alpha diversity of the roots in only one site (Bermuda; Supplementary Table S1). Thus, we grouped both the enriched and unenriched plots within a site to broadly examine regional variation in the seagrass microbiome.

For the seagrass microbial community analysis, a single, healthy-appearing *T. testudinum* shoot (extracted from the base of the shoot and inclusive of a small amount of rhizome) was harvested from the perimeter of each plot, rinsed with seawater to remove loosely attached sediment, and placed in a clean Ziplock bag. In the lab, 3 cm were cut from the basal portion of the epiphyte-free rank 2 leaf (second youngest leaf) with sterile scissors, and three root pieces were also collected with sterile plastic forceps. Adjacent to the location of the harvested shoot, a sterile syringe barrel was used to collect 2.5 ml of sediment from the surface. Three 750 μl samples of water were also collected from the surface per site with a sterile syringe barrel. All samples, including the 750 μl of water, were placed in tubes containing Xpedition Lysis/Stabilization Solution (ZYMO Research) to stabilize DNA until processing.

### DNA extractions, amplification, and sequencing

The Zymo Quick-DNA Fecal/Soil Microbe Microprep Kit (ZYMO Research) was used for DNA extraction. Once the DNA was extracted, samples were sent for Illumina MiSeq sequencing of the V4 region of the 16S rRNA gene. Illumina paired-end sequencing was done at the Environmental Sample Preparation and Sequencing Facility at Argonne National Laboratory (Chicago, IL, USA). DNA quantities were standardized by concentrating, diluting, and changing the sample volume to achieve normalization prior to sequencing. Primer set 515F-806R with adapters and barcodes for multiplexing were used. PCRs were run in 25 μl reactions: 9.5 μl of MO BIO PCR Water (Qiagen, Germantown, MD, USA), 12.5 μl of QuantaBio's AccuStart II PCR ToughMix (Quantabio, Beverly, MA, USA; 2 × concentration, 1 × final), 1 μl of forward primer (5 μM concentration, 200 pM final), 1 μl Golay barcode tagged reverse primer (5 μM concentration, 200 pM final), and 1 μl of template DNA. PCR conditions were as follows: initial denaturation at 94°C for 3 min, followed by 35 cycles of 94°C for 45 s, 50°C for 60 s, and 72°C for 90 s, and a final extension of 72°C for 10 min. After amplicons were quantified with PicoGreen (Invitrogen, Eugene, OR, USA) using a plate reader (Infinite^®^ 200 PRO, Tecan, Männedorf, Switzerland), samples were pooled in equimolar amounts and cleaned using AMPure XP Beads (Beckman Coulter, Indianapolis, IN, USA). A Qubit fluorometer (Qubit, Invitrogen, Eugene, OR, USA) was used to quantify the clean sample pool, which was then diluted to 2 nM, denatured, and diluted to 6.75 pM with 10% Phix spiked for Illumina MiSeq sequencing.

### Sequence processing

QIIME2 (v 2018.4.0; Bolyen et al., 2019) was used to demultiplex Illumina sequences, and R (v 4.2.1; R Core Team, 2013a) to run DADA2 (Callahan et al., 2016) to process the sequences and assign taxonomy using the SILVA release 132 database (Yilmaz et al., 2014). Amplicon Sequence Variants (99% ASVs) classified as “mitochondria” or “chloroplast” were filtered out of the data using the phyloseq package (McMurdie and Holmes, 2013) in R (v 4.2.1). ASVs with less than two counts in 25% of the samples were removed from the dataset as well. For the core microbiome study with multiple datasets, QIIME2 (v 2022.8) was used for classification using the SILVA release 132 database (Yilmaz et al., 2014). Raw sequences produced in this study are available at the NCBI Sequence Read Archive (accession number PRJNA1019313.

### Multi-study data processing

Prior studies that analyzed amplicon sequencing data generated from the same primer set (515F-806R) and that were of similar lengths (~250 bp) were combined with the amplicon sequencing data from this study for the analysis of the core microbiome of *T. testudinum*. Only studies on the *T. testudinum* leaf communities matched our criteria. These studies included Vogel et al. (2020, 2021a,b) and Rodríguez-Barreras et al. (2021). It is also important to note that three studies by Vogel et al. (2020, 2021a,b), used swabs to examine the microbial community of the leaf surface rather than the whole leaf, which might bias the results. The sites included for the comparisons are the six sites of the current study, Taylor Creek and Round Island in the Indian River Lagoon near Ft. Pierce, Florida, USA (Vogel et al., 2021a,b), Apalachee, Florida, USA (Vogel et al., 2020), and Cerro Gordo (Vega Baja, Puerto Rico), Isla de Cabra (Cataño, Puerto Rico), and Mar Azul (Luquillo, Puerto Rico; Rodríguez-Barreras et al., 2021).

Raw sequencing data from the selected studies were downloaded from the SRA archives (PRJNA691349, ERR4556266, ERR4556184, and ERR4556221). The sequence reads for the selected studies were already merged; thus, this study's paired-end reads were merged prior to combining all reads into a single file for processing. Briefly, the paired-end reads were merged using USEARCH v.11.0.667 (Edgar, 2010). The reads were merged if a ≥50 bp overlap was present, with a maximum of 5% mismatch. Reads with a maximum error rate of >0.001 or shorter than 200 bp were discarded. Primer sequences were trimmed using Cutadapt v.1.13 (Martin, 2011). The quality of the reads was assessed using FastQC v.0.11.8 (Andrews, 2010) and low-quality sequence ends were trimmed at a Phred quality (*Q*) threshold of 25 using a 10 bp sliding window in Sickle 1.33 (Joshi and Fass, 2011). After the removal of single sequence reads (using USEARCH), ASVs were identified using the UNOISE3 algorithm implemented in USEARCH with the default parameters, and an ASV table was generated using the otutab command. Taxonomy for the multi-study data was assigned using the Qiime2 qiime feature-classifier classify-sklearn command. Mitochondria and chloroplasts were filtered out of the dataset using the qiime taxa filter-table command. R (v 4.2.1; R Core Team, 2013a) was then used for further data analysis. The R packages microbiome (Lahti et al., 2017) and phyloseq (McMurdie and Holmes, 2013) were used to extract the core microbiome from the multi-study data set. Previous studies have defined core microbiomes as being present in 50%−100% of the samples, and as low as 30% (Neu et al., 2021). We considered ASVs that had at least two counts in at least 80% of the samples to form the core microbiome to avoid the excluding low abundance taxa, taking into account the wide range that spans between most of the sampling sites.

### Data analysis and visualization

R packages phyloseq (McMurdie and Holmes, 2013), ggplot2 (Wickham, 2009), vegan (Oksanen et al., 2018), stats (R Core Team, 2013b), pvclust (Suzuki and Shimodaira, 2006), qiime2R (Bisanz, 2018), tidyverse (Wickham et al., 2019), ape (Paradis and Schliep, 2019), viridis (Garnier et al., 2023), and ggordiplots (Quensen, 2020) were used for data visualization and microbial community analysis and statistics.

After removing outliers from the datasets, permutational multivariate analysis of variance (PERMANOVA) with 9,999 permutations was used to determine significant differences in the alpha-diversity among sites and region using the Chao1 and Shannon diversity metrics (Soriano-Lerma et al., 2020; Aires et al., 2021). Hierarchical clustering of the Euclidean distance matrices was used for analysis of the beta-diversity among the different sample types. Weighted Unifrac Distance Matrices were used for comparisons by sample type through ordination plots. A PERMANOVA analysis of the Bray-Curtis, Euclidean, Jaccard, Unifrac and Weighted Unifrac distance matrices was performed to determine whether there were significant differences in beta diversity by site or region. Factors were neither nested nor crossed as we were interested in the effects of sites and region alone.

To compare the taxonomic composition of microbial communities among sample types, the relative abundance of the top 20 genera were plotted with R (v 4.2.1). ASVs labeled “NA” were filtered out before determining the top 20 most abundant genera but were still considered for relative abundance calculations. The tax_glom function in phyloseq (McMurdie and Holmes, 2013) was used to combine all ASVs by assigned genus and then plotted with the plot_bar function in phyloseq. JMP pro (16.1.0, JMP^®^) was used for statistical analysis of the relative abundance of each of the top 20 genera by site (*n* = 6) using an analysis of variance (ANOVA) followed by a *post-hoc* Tukey's honest significant difference test (α = 0.05) and Bonferroni-adjusted *p*-values to correct for multiple genera.

## Results

### Alpha diversity

Shannon diversity indices of leaves and sediment microbial communities differed significantly by site (Table 1). Shannon diversity indices of the leaves, root, and water communities also varied significantly by region. Chao1 diversity of the roots, sediment, and water differed significantly by site, and varied significantly by region for the roots and water (Table 1).

**Table 1:** PERMANOVA of Chao1 and Shannon diversity indices to compare alpha diversity of microbial communities by sites and region.

**Sample-type**	**Site**		**Region**	
	**Chao1**	**Shannon**	**Chao1**	**Shannon**
Leaf	0.130	**0.004** ^**^	0.143	**0.011** ^*^
Root	**0.006** ^**^	0.261	**0.002** ^*^	**0.045** ^*^
Sediment	**0.001** ^**^	**0.002** ^**^	0.367	0.062
Water	**0.001** ^**^	0.092	**0.001** ^**^	**0.006** ^*^

Bold values and asterisks indicate significant differences.

^*^p < 0.05.

^**^p < 0.01.

**Figure 2:**
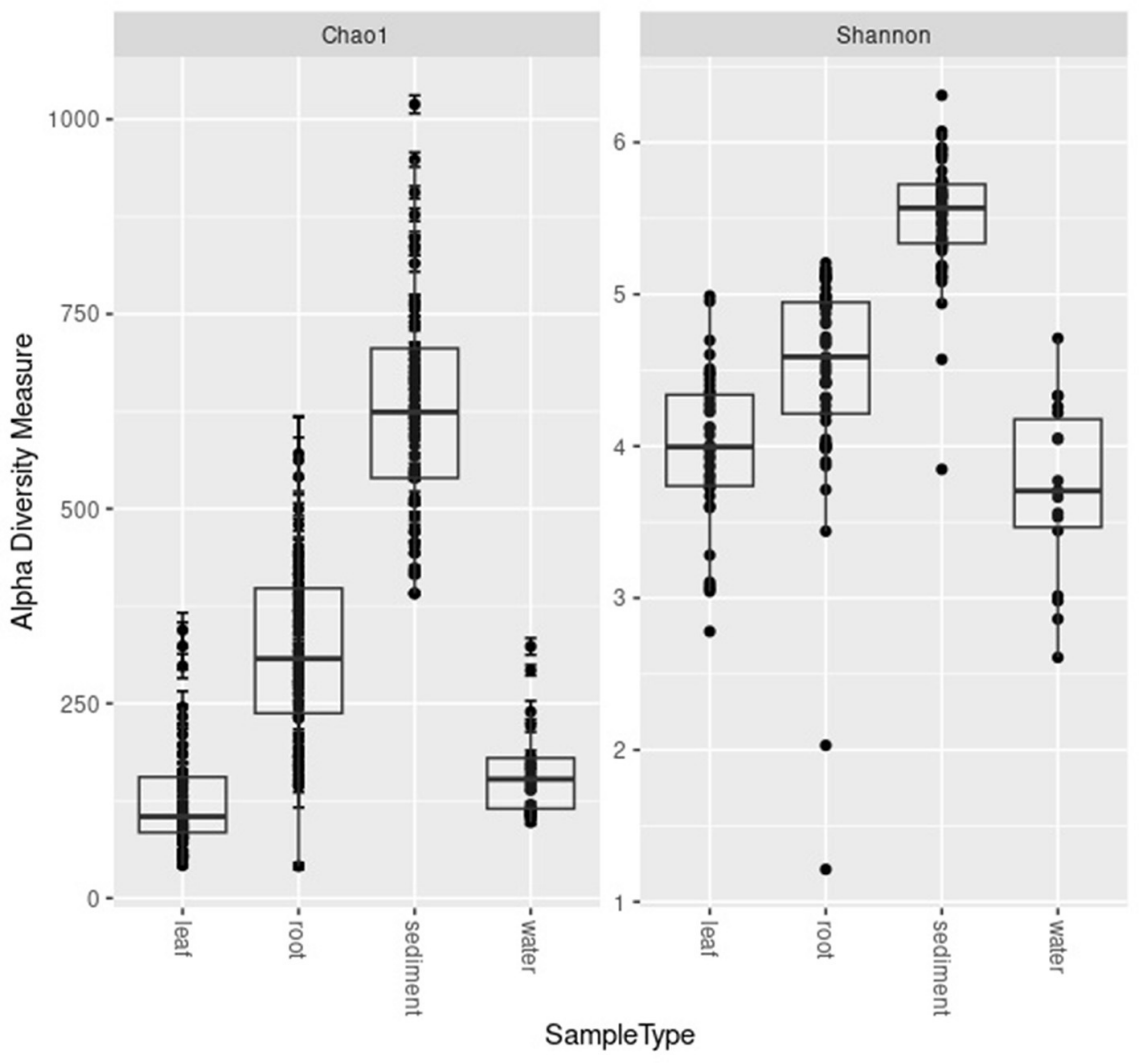
Boxplots of Chao1 and Shannon diversity metrics for the microbial communities of the leaves, roots, sediment, and water samples.

The highest alpha diversity was found in the sediment (average ± standard deviation for Chao1 634.45 ± 149.67 and for Shannon 5.56 ± 0.38; Figure 2). The alpha diversity of the microbial communities of the roots was the second highest (Chao1 398.97 ± 155.56; Shannon 4.71 ± 0.78) followed by the water samples (Chao1 173.61 ± 71.62; Shannon 3.76 ± 0.588) and finally the leaves (Chao1 112.58 ± 97.11; Shannon 3.75 ± 0.77; Figure 2).

### Beta diversity

Hierarchical clustering analysis suggested that the microbial communities clustered according to sample type (leaf, root, sediment, water; Supplementary Figure S1). Root and sediment communities always clustered more closely, suggesting more similarities in their microbial communities (Supplementary Figure S1). Leaves and water samples clustered more closely to each other at four of the six sites, while at Crystal River (FL, USA) and Bocas del Toro (Panama), leaves and water samples occurred in different branches in the cluster dendrograms, suggesting fewer similarities in their microbial communities compared to those clustering in the same branches (Supplementary Figure S1).

**Figure 3:**
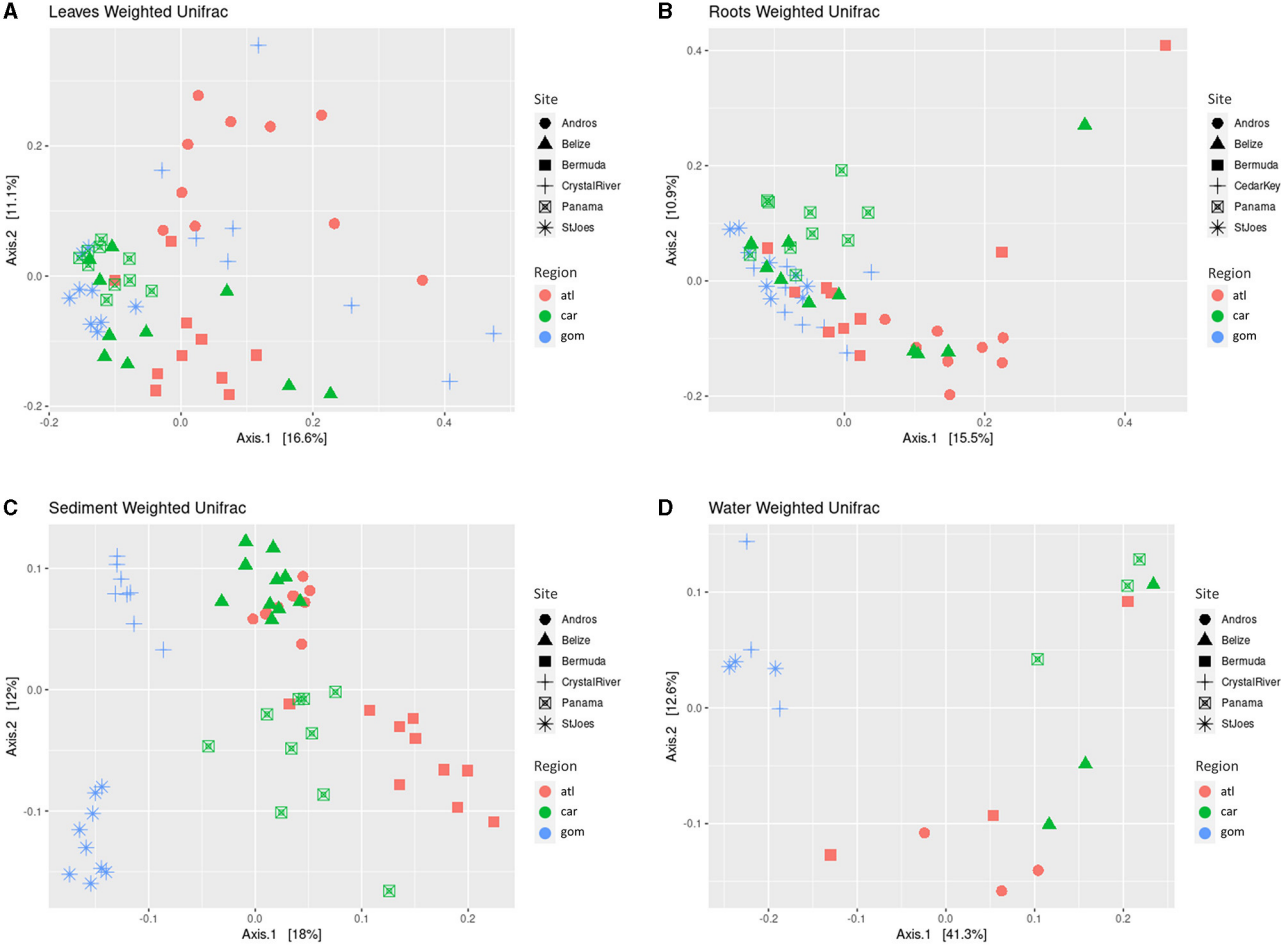
Principal coordinate analysis (PCoA) of weighted Unifrac distance matrices of the microbial communities of the leaves **(A)**, roots **(B)**, sediments **(C)**, and water samples **(D)**. Shapes depict sites. Colors indicate region. atl, Atlantic Ocean; car, Caribbean Sea; gom, Gulf of Mexico. Belize, Carrie Bow Cay (Belize); Bermuda, Riddell's Bay (Bermuda); Panama, Bocas del Toro (Panama); StJoes, St. Joseph Bay (FL, USA).

The beta diversity of the microbial communities of all sample types differed significantly by region and site, with the exception of water samples, which did not significantly differ by site (Table 2). Using the Weighted Unifrac Distance metric to visualize clustering based on similarities, the sediment and water communities cluster more distinctly by region as compared with plant-associated sample types, with Crystal River (FL, USA) and St. Joseph Bay (FL, USA) being the most distinct, while the remaining sites cluster more closely together (Figure 3). The differences in sediment communities might be driven by region (Gulf of Mexico vs. Atlantic and Caribbean; Figure 3C) as well as site. The water communities seem to cluster mostly by region (Figure 3D) and the Gulf of Mexico water samples were the most distinct, while Caribbean Sea and Atlantic Ocean samples were more similar, but still mostly cluster separately (Figure 3D).

**Table 2:** PERMANOVA results for the comparison of the Bray-Curtis, Euclidean, and Jaccard beta diversity metrics of the microbial communities of the leaves, roots, sediment, and water samples.

**Sample type**	**Bray-Curtis**	**Euclidean**	**Unifrac**	**Weighted unifrac**	**Jaccard**
**Site**					
Leaf	0.0001^***^	0.0001^***^	0.0001^***^	0.0001^***^	0.0001^***^
Root	0.0001^***^	0.0001^***^	0.0001^***^	0.0001^***^	0.0001^***^
Sediment	0.0001^***^	0.0001^***^	0.0001^***^	0.0001^***^	0.0001^***^
Water	0.0001^***^	0.085^****^	0.0001^***^	0.0001^***^	0.0001^***^
**Region**					
Leaf	0.0001^***^	0.0005^***^	0.0001^***^	0.0001^***^	0.0001^***^
Root	0.0001^***^	0.0001^***^	0.0001^***^	0.0001^***^	0.0001^***^
Sediment	0.0001^***^	0.0001^***^	0.0001^***^	0.0001^***^	0.0001^***^
Water	0.0001^***^	0.0124^*^	0.0002^***^	0.0001^***^	0.0001^***^

^*^p < 0.05.

^***^p < 0.001.

^****^p < 0.1.

When comparing the Weighted Unifrac distance of the leaves and roots, no clear, distinct clustering by region is apparent as samples mostly cluster together, with slight distinctions by site (Figures 3A, B). Despite differing significantly in several beta diversity metrics (Bray-Curtis, Euclidean, Jaccard, Unifrac, and Weighted Unifrac; Table 2), the clustering from the Weighted Unifrac distance metric suggests that some similarity still occurs among the microbial communities of all sites when considering both the presence and the abundance of taxa in the leaves and in the roots, suggesting a core microbiome is present.

### Multi-study community analysis and core microbiome

**Figure 4:**
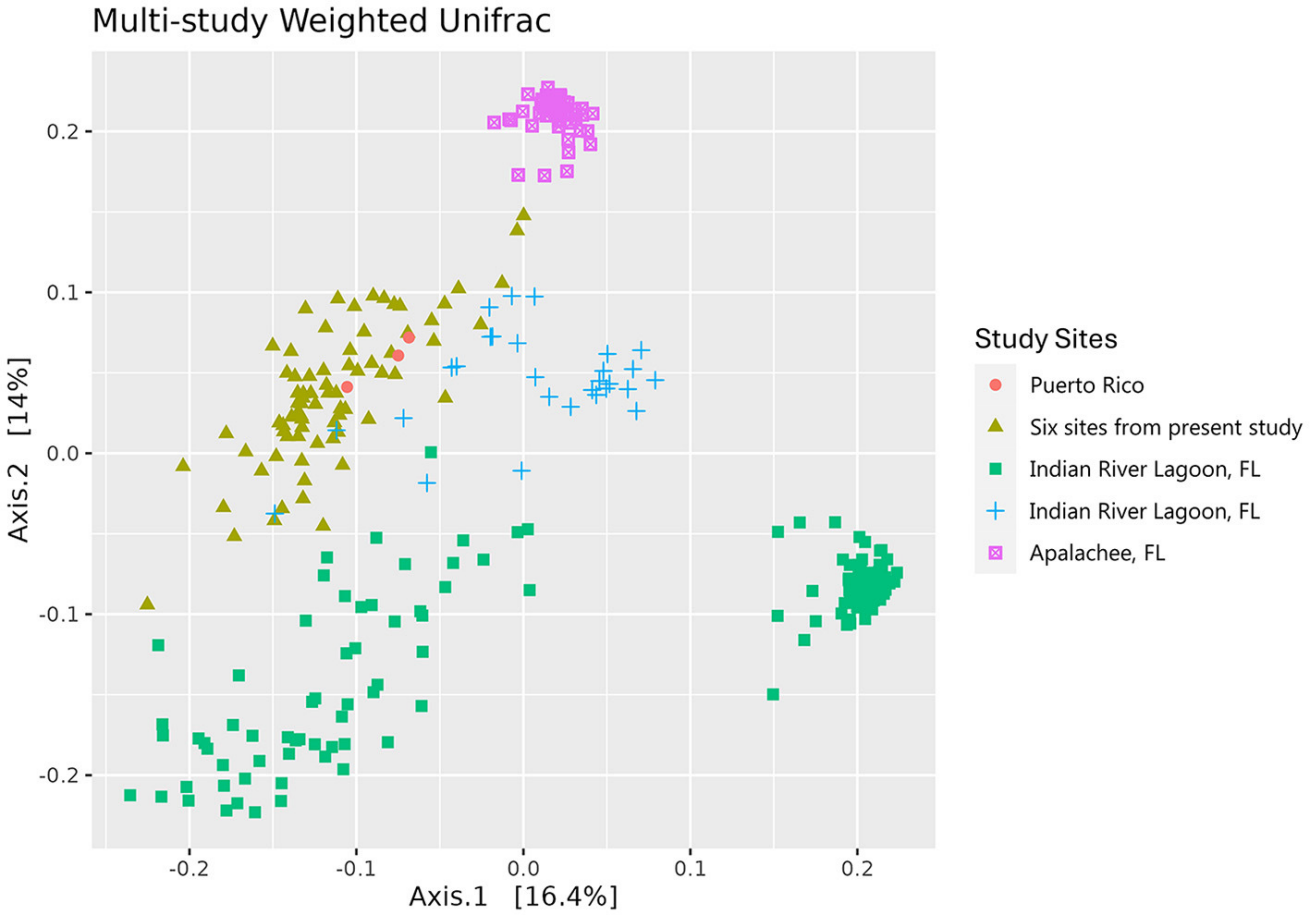
Principal coordinate analysis (PCoA) of the weighted Unifrac distance matrices of the microbial communities of the leaves of *Thalassia testudinum* from multiple studies. Colors and shapes indicate the sites covered by different studies: peach circles = Puerto Rico (Rodríguez-Barreras et al., 2021), mustard triangles = Andros, St. Joseph Bay, Crystal River, Riddell's Bay, Bocas del Toro, Carrie Bow Cay (present study), green squares = Indian River Lagoon, FL (Vogel et al., 2021a), blue crosses = Indian River Lagoon, FL (Vogel et al., 2021b), magenta square saltires = Apalachee, FL (Vogel et al., 2020).

The Weighted Unifrac distance metric of the leaf communities of all included studies (Vogel et al., 2020, 2021a,b; Rodríguez-Barreras et al., 2021) were similar in most field collected samples, with the exception of the Apalachee, Florida samples (Vogel et al., 2020), which form a distinct cluster (Figure 4). The study on *T. testudinum* plants that were transplanted into aquaria from the Indian River Lagoon are separated into two clusters: one that is closely similar to the microbiome of most field collected samples and one that forms a more distinct cluster (Vogel et al., 2021a). It is possible that the former are the samples that were taken after 10 days of acclimation, while the latter are the ones taken at the end of the 30-day experiment, resulting in an altered surface microbiome (Vogel et al., 2021a).

There were 14 ASVs that comprised the core microbiome (Supplementary Table S2). Three of these were classified to genus level, including *Labrenzia, Rhodovulum, Methylotenera*, and one was classified to species: *Hirschia maritima*. The remaining ASVs were only classified to family level: Rhodobacteraceae (eight ASVs), Halieaceae, and Hyphomonadaceae (two ASVs; Supplementary Table S2). All core microbiome ASVs were present in all studies except for four ASV (belonging to the family Rhodobacteraceae) that were absent in Rodríguez-Barreras et al. (2021) (Zotu152, Zotu16, Zotu343, Zotu99). ASV Zotu78 had the highest average relative abundance (0.4%−8.7%) within the core microbiome, while Zotu343 had the lowest (0–0.58%, Supplementary Table S2). In general, most ASVs in the core microbiome belong to the family Rhodobacteraceae (eight ASVs), while a few belong to Hyphomonadaceae (three ASVs). The remaining taxa of the core microbiome only had one ASV in the families Stappiaceae, Halieaceae, and Methylophilaceae. The sequences for these ASVs are available in the Supplementary material.

### Abundant taxa

**Figure 5:**
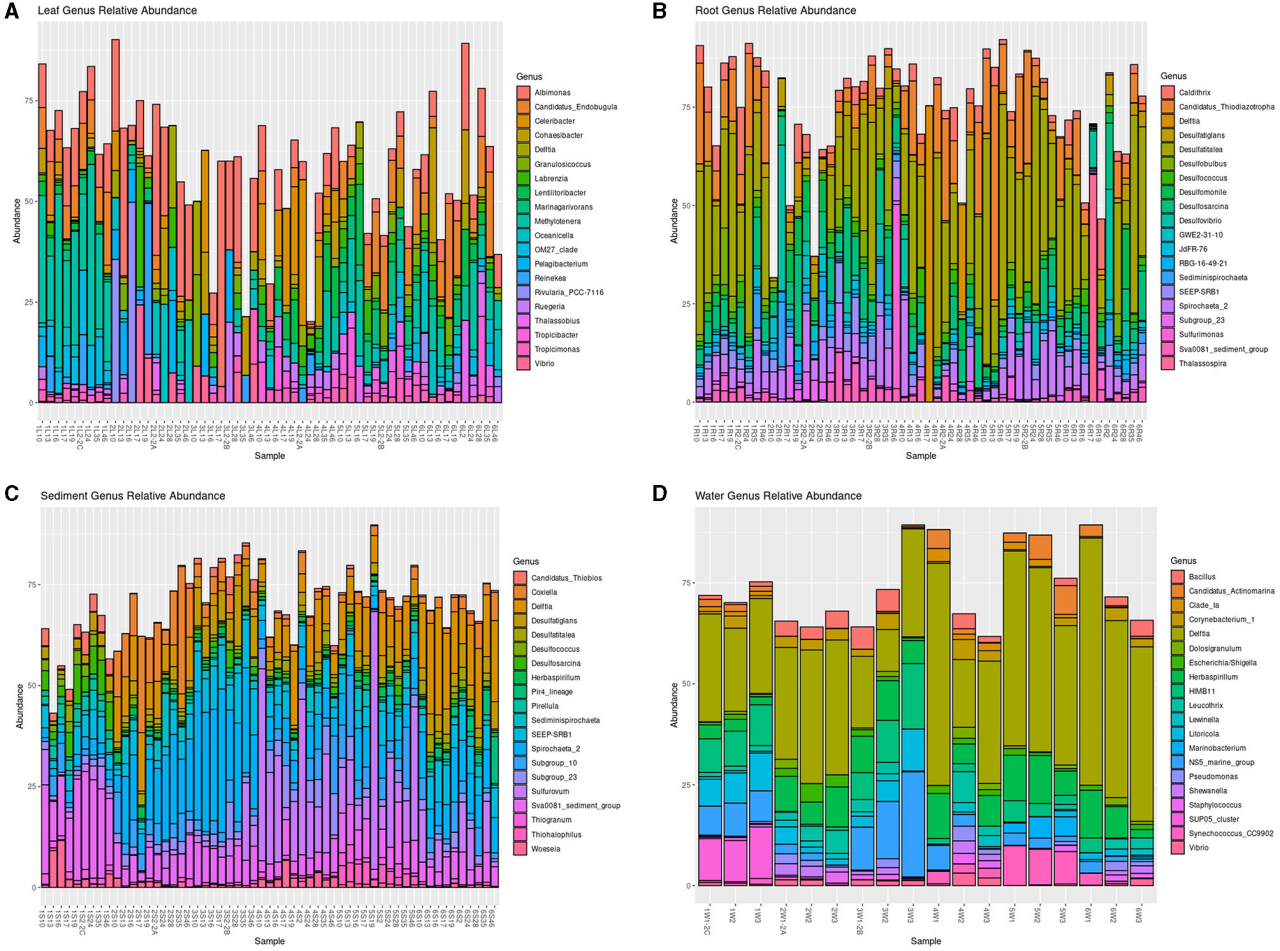
Top 20 most abundant taxa of the microbial communities of the leaves **(A)**, roots **(B)**, sediment **(C)**, and water **(D)** samples. Genera labeled NA were filtered out of dataset. Sample names that start with 1 are St. Joseph Bay, 2 Andros, 3 Crystal River, 4 Riddell's Bay, 5 Bocas del Toro, and 6 Carrie Bow Cay.

The relative abundance of the top 20 genera per sample type (leaves, roots, sediment, and water) are shown in Figure 4. Several genera were abundant in more than one sample type, including *Delftia* in all sample types; *Vibrio* in the leaves and water; *Desulfatiglans, Desulfatitalea, Desulfococcus, Desulfosarcina, Sediminispirochaeta, SEEP-SRB1, Spirochaeta 2, Subgroup 23*, and *Sva0081* in the roots and sediment; and *Herbasipillum* in the sediment and water (Figure 5, Supplementary Tables S3–S6). The most abundant genera seem to be present in most samples within each sample type and have differential prevalence among the sites.

#### Leaves

The leaf communities were similar among sites, with 13 of the top 20 genera present in all sites, except for seven genera: *Celeribacter* [absent in Carrie Bow Cay (Belize) and Bocas del Toro (Panama)], *Lentilitoribacter* [absent in Andros (Bahamas) and Crystal River (FL, USA)], *Pelagibacterium* [absent in Bocas del Toro (Panama)], *Rivularia_PCC-7116* [absent in Riddell's Bay (Bermuda), Crystal River (FL, USA), Bocas del Toro (Panama), and St. Joseph Bay (FL, USA)], and *OM27_clade, Thalassobius*, and *Tropicibacter* [absent in Crystal River (FL, USA); Figure 5A, Supplementary Table S3]. Several genera were significantly more abundant in certain sites: *Candidatus* Endobugula in Carrie Bow Cay (Belize), Bocas del Toro (Panama), and St. Joseph Bay (FL, USA; *p* = 0.002); *Celericabter* and *Lentilitoribacter* in Riddell's Bay (Bermuda; *p* = 0.004 and *p* = 0.018, respectively); *Marinagarivorans* in Bocas del Toro (Panama; *p* = 0.002); *Methylotenera* and *OM27_clade* in St. Joseph Bay (FL, USA; *p* = 0.002 for both), and *Oceanicella* in Andros (Bahamas; *p* = 0.046). The remaining genera had no significant differences in their relative abundance by site.

#### Roots

The root communities were similar with most of the top 20 genera present in all sites except for *Thalassospira*, which was only present in Carrie Bow Cay (Belize) and St. Joseph Bay (FL, USA; Figure 5B, Supplementary Table S4). Seven of the 20 genera differed significantly in their relative abundance between sites. *Candidatus* Thiodiazotropha was significantly more abundant in Bocas del Toro (Panama) than in the other sites (*p* = 0.016); *Desulfatitalea* was significantly more abundant in Bocas del Toro (Panama) and St. Joseph Bay (FL, USA; *p* = 0.01); *GWE2-31-10, RBG-16-49-21*, and *Subgroup_23* were significantly more abundant in Andros (Bahamas; *p* = 0.024, *p* = 0.002, and *p* = 0.002, respectively); *SEEP-SRB1* and *Sva0081_sediment_group* were significantly more abundant in Crystal River (FL, USA; *p* = 0.002, for both; Supplementary Table S4).

#### Sediment

All top 20 genera except for one genus, *Thiohalophilus* (absent in Crystal River), were present in all sites (Figure 5C; Supplementary Table S5). All but two genera, *Sediminispirochaeta* and *Subgroup_10*, differed significantly in their abundance by site (Supplementary Table S5). *Candidatus_*Thiobios is significantly more abundant in St. Joseph Bay (FL, USA), followed by Crystal River (FL, USA; *p* = 0.002); *Coxiella* in Andros (Bahamas; *p* = 0.002); *Delftia* in Carrie Bow Cay (Belize), followed by Andros (Bahamas), Riddell's Bay (Bermuda) and Bocas del Toro (Panama; *p* = 0.002); *Desulfococcus* and *Desulfosarcina* in St. Joseph Bay (FL, USA; *p* = 0.002 for both); *Herbaspirillum, Thiogranum*, and *Thiohalophilus* in Bocas del Toro (Panama; *p* = 0.002 for all); *Pir4_lineage* in Belize (*p* = 0.002); *Pirellula, Sva0081_sediment_group*, and *Woeseia* in St. Joseph Bay (FL, USA; *p* = 0.002); *SEEP-SRB1* and *Spirochaeta_2* in Crystal River (FL, USA; *p* = 0.002 for both); *Subgroup_23* and *Sulfurovum* in Riddell's Bay (Bermuda; *p* = 0.002 and *p* = 0.008, respectively; Supplementary Table S5).

#### Water

Water communities were similar between sites with 16 of the top 20 genera present in all sites. *Candidatus_*Actinomarina and *Clade_Ia* were absent in Andros (Bahamas); *Marinobacterium* was absent in Andros (Bahamas), Riddell's Bay (Bermuda), and St. Joseph Bay (FL, USA); and *SUP05_cluster* was only present in Crystal River (FL, USA; Figure 5D; Supplementary Table S6). Seven of the top 20 genera differed significantly by site: *Clade_Ia* was significantly more abundant in Riddell's Bay (Bermuda) and Bocas del Toro (Panama; *p* = 0.008); *HIMB11* and *NS5_marine_group* in Crystal River (FL, USA; *p* = 0.006 and *p* = 0.026, respectively); *Litoricola* and *SUP05_cluster* in St. Joseph Bay (FL, USA; *p* = 0.028 and *p* = 0.002, respectively); and *Marinobacterium* and *Synechococcus_CC9902* in Bocas del Toro (Panama; *p* = 0.002 for both; Supplementary Table S6).

## Discussion

### Microbial communities by site and region

The microbial communities of *T. testudinum* in this study differ by sample type (water, sediment, leaves, roots) with high similarities between sediment and roots and partially between the leaves and the water samples. Significant differences in the microbial communities between sites, both in alpha and beta diversity, were also observed. Several studies on various seagrass species corroborate these results (Cúcio et al., 2016; Fahimipour et al., 2017; Rotini et al., 2017; Crump et al., 2018; Hurtado-McCormick et al., 2019; Ugarelli et al., 2019). Our data also indicate that the microbial communities differ by region (i.e., Atlantic Ocean, Caribbean Sea, or Gulf of Mexico), with alpha and beta diversities differing significantly for all sample types; however, the weighted Unifrac distances of the leaf and root communities do not show distinct clustering, suggesting that the plant-associated microbial communities of *T. testudinum* share some, although not statistically significant similarities between regions (Figure 3; Tables 1, 2). This further suggests that a core microbiome is present at least in the leaves and root samples. Water and sediment samples show more apparent clustering by region, suggesting a clearer distinction in the microbial communities of the surrounding environments. Interestingly, sediment communities were impacted by region only under the Shannon diversity metric (i.e., richness and evenness) and not the Chao1 metric (i.e., richness), suggesting that species evenness, rather than richness, is driving the differences in the sediment microbial communities (Table 1). The significant differences in the microbial communities between regions is likely due to the diverse physicochemical parameters at each location (e.g., pH, salinities, nutrient levels of the water and sediment), which have been shown to affect the communities of seagrasses in other studies (Hassenrück et al., 2015; Vogel et al., 2020, 2021a; Wang L. et al., 2020; Banister et al., 2021). Furthermore, evidence suggests that the microbial communities of seagrasses can differ temporally by season (Korlević et al., 2021) as well as time of day (Rotini et al., 2020; reviewed in Conte et al., 2021a). Because the samples collected from each site at each region were not collected on the same day nor at exactly the same time of day, we cannot exclude that the differences in the microbiomes can also be due to temporal variations.

Previous studies have shown that sediment lithology can influence the microbial communities of sediments throughout various aquatic systems, including marine (Hoshino et al., 2020), deep Arctic (Kanzog and Ramette, 2009), rivers (Wang et al., 2016), and lagoons (Obi et al., 2016; Aldeguer-Riquelme et al., 2022), possibly due to the composition of organic matter. In seagrass meadows, several factors were shown to significantly impact the microbial communities of the sediments, including carbon (C) to N ratios as well as a combination of total inorganic C, total organic C, dissolved oxygen, pH, and seagrass density (*Z. marina*, Ettinger et al., 2017), in addition to location, plant nutrient composition, sediment composition (e.g., moderate-fine sandy soil and silt-sandy soil), and sediment grain size (*T. hemprichii, Enhalus acoroides*, or a mix of both) (Zhang et al., 2020, 2022). Despite their importance in determining the microbial communities of sediments in seagrass meadows, few studies have evaluated the impact of sediment parameters on seagrass-associated microbiomes. In the present study, there were three sites with high carbonate sediment, and one site for the intermediate carbonate, mixed carbonate, and low carbonate (siliciclastic) sediment types. Although the sample size was not large enough to examine the effects of sediment type on the microbial communities of *T. testudinum*, it is also possible that this factor played a role in shaping the microbial communities (Supplementary Figure S2). More sampling sites for each sediment type would provide a better scope of the effects of sediment composition on the *T. testudinum* microbiome.

### Core microbiome across regions and studies

To date, the only large-scale study on the microbiome associated with the leaves, roots, sediment, and water samples reports on *Z. marina* meadows; this study covers most of its geographical range, which is more extensive than the range of *T. testudinum* (Fahimipour et al., 2017). No such study is available for other seagrass species. Fahimipour et al. (2017) found that the microbiome differed by site and sample type, and they found evidence of a core microbiome, at least on the roots. The authors also found more distinct differences in their leaf communities and that the communities of the leaves were similar to that of the water. This is not generally the case in other species like *H. ovalis* and *P. australis* (Roth-Schulze et al., 2016), nor *T. testudinum* (Ugarelli et al., 2019; Vogel et al., 2020), where the leaf communities differ clearly from the water communities. This indicates a strong distinction between the microbiome composition of the two seagrass species, where it seems that *T. testudinum* has a more defined leaf-associated core microbiome in most of its geographical range. Aires et al. (2021) also suggests a more distinct core microbiome in *T. testudinum* compared to *H. stipulacea* and *H. wrightii* in the Caribbean Sea.

In the present study, while the microbial communities of all sample types differed significantly, Weighted Unifrac PCoA plots show some similarities in microbial community compositions of the leaves and roots across regions (Figure 3). This indicates that, although the microbial communities significantly differ between sites in presence-absence and abundances, a core microbiome composed of similar taxa is likely still present in *T. testudinum* plants across the three regions. Indications of core microbiomes have also been found in other seagrass studies on various species (Cúcio et al., 2016; Bengtsson et al., 2017; Hurtado-McCormick et al., 2019; Vogel et al., 2020), and it has been suggested that seagrasses might actually select for a core microbiome (Roth-Schulze et al., 2016; Kardish and Stachowicz, 2021). The concept of the core microbiome ties in with the holobiont concept, which suggests that plants and their microbial communities act as one functional unit to ensure the survival of the whole system (Lemanceau et al., 2017; Jones et al., 2019). For example, in *Agarophyton* seaweed, surface metabolites were shown to deter pathogens while attracting microbes with a potential to protect against disease, i.e., host and microbes work together to prevent disease, ultimately benefiting the holobiont (Saha and Weinberger, 2019). The core microbiome is usually defined as microbial taxa that are present in the microbial communities of several members of the same species and are not necessarily abundant (Lundberg et al., 2012; Neu et al., 2021). In general, studies have defined core microbiomes as being present in 50%−100% of the samples, and as low as 30% (Neu et al., 2021). However, most studies require OTUs to be present in 100% of the samples, which can risk the exclusion of low-abundance taxa that can be of importance (Neu et al., 2021). For our purposes, we defined the leaf core microbiome as taxa that were present in at least 80% of the samples to avoid the exclusion of low abundance taxa, especially because of the wide range that spans between most of the sampling sites. It is debated whether core microbiomes are functional, meaning different taxa serve the same function in the community across different plants of the same species, or taxonomical, meaning the same taxa are present to serve the same function in plants of the same species (Lemanceau et al., 2017; Jones et al., 2019; Neu et al., 2021). In this study, it seems likely that the core microbiome of *T. testudinum* across the Caribbean might be in part taxonomical as well as functional.

The results of this study are unique in that they suggest similarities in the microbial communities of plant associated microbes at the genus level throughout a wide geographical range of *T. testudinum* that includes sites that are thousands of kilometers apart, while other studies usually focus on shorter ranges (e.g., Vogel et al., 2020, 2021b; Aires et al., 2021). Combining the amplicon data that is available from *T. testudinum* studies allows a better investigation of the plant associated core microbiome of *T. testudinum*. After combining our amplicon data with amplicon data that are similar in sequence length and were generated with the same primers used in this study (Vogel et al., 2020, 2021a,b; Rodríguez-Barreras et al., 2021), we found strong similarities in field collected *T. testudinum* leaf microbiomes, suggesting a leaf core microbiome exists in *T. testudinum*. This is important because it suggests that *T. testudinum* houses a core microbiome that is widespread throughout most of its geographical range, and possibly even all of its range, at least in the phyllosphere communities.

The multi-study core microbiome consisted of 14 ASVs, which is within the range of prior core microbiome studies [21 ASVs in Vogel et al., 2020 (*T. testudinum*), six ASVs in Hurtado-McCormick et al., 2019 (*Z. muelleri*), and 14 ASVs in Bengtsson et al., 2017 (*Z. marina*)] but at a much wider geographical span than previous studies. Out of the 14 core ASVs, 8 ASVs are in the family Rhodobacteraceae, which is known to produce secondary metabolites that have antimicrobial, quorum sensing, and iron chelating functions (Henriksen et al., 2022). This family also contains purple non-sulfur bacteria and has members that are involved with the biogeochemical cycles of sulfur and carbon (Pujalte et al., 2014). Rhodobacteraceae have also been found in other seagrass species as part of the core microbiome, such as *T. hemprichii* (Rotini et al., 2020), *Z. muelleri* (Hurtado-McCormick et al., 2020), and *Z. marina* (Bengtsson et al., 2017). In the core microbiome of *T. testudinum*, we also found three ASVs in the family Hyphomonadaceae. This family is composed of prosthecate bacteria that produce a polysaccharide-based adhesin that allows for primary colonization of surfaces and biofilm formation (Abraham and Rohde, 2014; Oberbeckmann et al., 2018). Hyphomonadaceae were found as part of the core microbiome of the leaves of *T. hemprichii* (Rotini et al., 2020). The core microbiome also included one ASV in the family Stappiaceae, one in Halieaceae, and one in Methylophilaceae. Certain genera within Stappiaceae, such as *Stappia*, can oxidize carbon monoxide (Weber and King, 2007), and the family has been found associated with *H. stipulaceae* roots (Conte et al., 2023). Halieaceae have been previously found in *Cymodocea nodosa* sediments (Markovski et al., 2022), but not on the leaves. This family includes genera involved with photoheterotrophy (Spring et al., 2015). Finally, Methylophilaceae was also found as part of the leaf core microbiome of *Z. marina* (Bengtsson et al., 2017; Adamczyk et al., 2022), and are known as methylotrophs, capable of using methanol and methylamine as energy sources (Doronina et al., 2014).

Although this study was able to compare the leaf microbiome data from four previous studies, several other *T. testudinum* leaf and root microbiome data exist, but the methods employed for sampling and data processing differed from this study and were therefore not included in the multi-study core microbiome analysis. Without standardized sampling and data processing methods, proper data comparisons are difficult. Furthermore, to have a better scope of the composition of the core microbiome of *T. testudinum*, more sampling locations should be included for the leaves and similar studies should also be performed on the roots when more comparable data is available. With a more defined core microbiome, these studies can later lead to investigating the functions that the core microbiome serves on *T. testudinum* and possibly ways to monitor the health of seagrass meadows.

### Abundant taxa include genera with potentially beneficial functions for the host

In general, several genera with potentially important functions were abundant in the microbiome of *T. testudinum* throughout all our sampling locations. Nitrogen fixers such as *Delftia* (Agafonova et al., 2017) were found in all sample types. Other N-fixers were found only in certain sample types, such as *Candidatus* Thiodiazotropha (Petersen et al., 2016) in the roots, *Herbaspirillum* (Ureta et al., 1995), in the water and sediment samples, and *Desulfatitalea* (Rolando et al., 2022) in the roots and sediment. *Candidatus* Thiodiazotropha not only fixes N but can also oxidize sulfur (Rolando et al., 2022). In other seagrass species, such as *H. ovalis, Candidatus* Thiodiazotropha was associated with stressed plant roots (Martin et al., 2019) and this genus is also commonly found in lucinid bivalves that are associated with seagrasses (König et al., 2016). Interestingly, our findings show that *Candidatus* Thiodiazotropha was significantly less abundant on the roots of Andros (Bahamas), where the leaf N:P ratios were significantly higher than the other sites (Fourqurean et al., 2023; Supplementary Table S4). It is possible that this genus is less prevalent at this site because the plants have more nitrogen relative to phosphorus (P) in their leaves and likely more P-limited relative to other sites. A similar case might be true for *Desulfatitalea*, which is both an N fixer and a sulfate reducer (Higashioka et al., 2013; Rolando et al., 2022) and was significantly less abundant in the roots of Andros (Bahamas) compared to the roots of Bocas del Toro (Panama) and St. Joseph Bay (FL, USA), and relatively less abundant than the remaining sites (Supplementary Table S4). This genus was found in the roots of *T. testudinum* in previous studies (Aires et al., 2021), and in other seagrasses and saltwater plants, such as *H. wrightii* and *H. stipulacea* (Aires et al., 2021), and *Spartina alterniflora* (Rolando et al., 2022).

Other abundant taxa with potential benefits for *T. testudinum* include *Delftia*, which can promote plant growth, antagonize pathogens, and fix N (Agafonova et al., 2017), and was present in all sample types (Figure 5). *Labrenzia* displayed antimicrobial activity against bacteria and fungi (Amiri Moghaddam et al., 2018; Raj Sharma et al., 2019), can form biofilms (Zaynab et al., 2021), and was present in the leaves of all the sites. *Celeribacter*, which is known to contain genes for import and export of C, N, P, and sulfur (S) (Wang X. et al., 2020) and potentially provides some of these nutrients to the plant, was present in the leaves of all sites, except for the Caribbean Sea sites (Supplementary Table S3). *Celeribacter* was previously detected in *H. stipulacea* and *T. testudinum* (Aires et al., 2021) as well. *Candidatus* Endobugula was most abundant in the leaves of the Caribbean Sea sites and in St. Joseph Bay (FL, USA) (Supplementary Table S3), and, in bryozoans, are known to produce bryostatins and polyketides, which have been shown to chemically deter fish from eating their larvae (Davidson et al., 2001; Lim and Haygood, 2004; Lopanik et al., 2004). *Ruegeria* was also prevalent in the leaves of all sites and has previously been associated with possibly producing probiotics for coral (Kitamura et al., 2021), containing algicidal properties (Riclea et al., 2012), and has previously been found in seagrasses (Weidner et al., 2000). In the root samples, *JdFR-76* has been correlated with suppression of root rot disease in oil palms (Goh et al., 2020), and possibly can also aids in disease suppression for *T. testudinum*. Several sulfur oxidizers were also present in the roots of *T. testudinum* including *Candidatus* Thiodiazotropha, *Spirochaeta 2* (Dubinina et al., 2011), and *Sulfurimonas*, which has been linked with seagrass rhizospheres in *Enhaulus acoroides* (Zhang et al., 2022). Sulfur oxidizers can potentially help seagrasses withstand sulfide, which is toxic to plants, by oxidizing it to sulfate (reviewed in Ugarelli et al., 2017).

Within the top 20 most abundant taxa, there were also taxa that can be associated with diseases in marine flora and fauna, and possible common human pathogenic genera in the water samples. In the leaves, some of these genera include *Cohaesibacter*, associated with stony coral tissue loss disease (Rosales et al., 2020), *Granulosicoccus*, associated with black/purple tissue in *Z. muelleri* (Hurtado-McCormick et al., 2020), and *Thalassobius*, associated with lobster epizootic shell disease (Quinn et al., 2012). In the roots, *SEEP-SRB1* and *Desulfomonile* have been previously linked to stressed *H. ovalis* roots (Martin et al., 2020) and are known as sulfate reducers. Other sulfate reducers found in the roots of *T. testudinum* include *Desulfatiglans* (Galushko and Kuever, 2019), *Desulfobulbus* (Higashioka et al., 2013), *Desulfococcus* (Bridge et al., 1999), *Desulfosarcina* (Kleindienst et al., 2014), and *Desulfovibrio* (Price et al., 2014). The water samples contained taxa that can be associated with human pathogens, including *Corynebacterium, Escherichia/Shigella*, and *Staphylococcus*. Further information on the possible functions and/or interesting facts of the top 20 most abundant taxa can be found in Supplementary Table S7.

## Conclusion

In conclusion, this study shows that *T. testudinum* microbiomes significantly differ across a large geographical range by site and region, and that potentially beneficial taxa are present in their microbiomes. Most importantly, this study shows that *T. testudinum* shares a leaf core microbiome that is present, not only in these six sites and three regions, but also in other studies that included sites in Taylor Creek and Round Island in the Indian River Lagoon near Ft. Pierce, Florida, USA (Vogel et al., 2021a,b), Apalachee, Florida, USA (Vogel et al., 2020), and Cerro Gordo in Vega Baja, Puerto Rico, Isla de Cabra in Cataño, Puerto Rico, and Mar Azul in Luquillo, Puerto Rico (Rodríguez-Barreras et al., 2021). Knowing the composition of the core microbiome of *T. testudinum* can provide us with a tool that we can use to monitor seagrass health by determining patterns of changes in the core microbiome composition of stressed vs. healthy plants.

## Data availability statement

The data presented in the study were deposited in the NCBI Sequence Read Archive repository, accession number PRJNA1019313.

## Author contributions

KU: Writing – review & editing, Data curation, Formal analysis, Visualization, Writing – original draft. JC: Writing – review & editing, Conceptualization, Funding acquisition, Methodology, Resources, Supervision. OR: Resources, Writing – review & editing. CJM: Resources, Writing – review & editing. AA: Resources, Writing – review & editing. JD: Resources, Writing – review & editing. KH: Resources, Writing – review & editing. VP: Resources, Writing – review & editing. SB: Resources, Writing – review & editing. LC: Resources, Writing – review & editing. JF: Resources, Writing – review & editing. TF: Writing – review & editing. SL: Writing – review & editing, Resources. CWM: Resources, Writing – review & editing. AM: Resources, Writing – review & editing. VM: Resources, Writing – review & editing. SM: Resources, Writing – review & editing. CM-M: Resources, Writing – review & editing. LKR: Resources, Writing – review & editing, Supervision. AR: Resources, Writing – review & editing. LMR: Resources, Writing – review & editing. YS: Resources, Writing – review & editing. KS: Resources, Writing – review & editing. WW: Resources, Writing – review & editing. CC: Investigation, Methodology, Visualization, Writing – review & editing, Data curation, Formal analysis. US: Writing – review & editing, Conceptualization, Project administration, Resources, Supervision.

## Supplementary material

Supplementary Figure S1 Cluster Dendrogram of the Euclidean distance matrices of the microbial communities of the leaf, root, sediment, and water samples of each site. Sediment is orange, roots are brown, water is blue, and leaves are green.

Supplementary Figure S2 Principal coordinate analysis (PCoA) of weighted Unifrac distance matrices of the microbial communities of the leaves **(A)**, roots **(B)**, sediments **(C)** and water samples **(D)**. Shapes depict sites. Colors indicate sediment-type. atl, Atlantic Ocean; car, Caribbean Sea; gom, Gulf of Mexico. H-carb, high carbonate; I-carb, intermediate carbonate; L-carb_sil, low carbonate (siliciclastic); M-carbsil, mixed carbonate siliciclastic.

Supplementary Table S1 PERMANOVA of both alpha (Shannon and Chao1) and beta (Bray-Curtis and Euclidean) diversity indices to compare the microbial community composition between enriched and non-enriched plots at each site.

Supplementary Table S2 Relative abundance and taxonomic data of ASVs that compose the core microbiome of *T. testudinum* across several studies.

Supplementary Table S3 ANOVA results of the relative abundance of the Top 20 most abundant leaf genera by site, with Bonferroni corrected *p*-values, followed by *post-hoc* Tukey's HSD.

Supplementary Table S4 ANOVA results of the relative abundance of the Top 20 most abundant root genera by site, with Bonferroni corrected *p*-values, followed by *post-hoc* Tukey's HSD.

Supplementary Table S5 ANOVA results of the relative abundance of the Top 20 most abundant sediment genera by site, with Bonferroni corrected *p*-values, followed by *post-hoc* Tukey's HSD.

Supplementary Table S6 ANOVA results of the relative abundance of the Top 20 most abundant water genera by site, with Bonferroni corrected *p*-values, followed by *post-hoc* Tukey's HSD.

Supplementary Table S7 Top 20 most abundant genera by sample type with a list of possible functions and/or interesting facts relating to seagrasses, marine flora or fauna, or other plant systems.
