# Supplementary material for: Microbiomes of Thalassia testudinum throughout the Atlantic Ocean, Caribbean Sea, and Gulf of Mexico are influenced by site and region while maintaining a core microbiome
Source: Front Microbiol. 2024 Feb 23;15:1357797. doi: 10.3389/fmicb.2024.1357797 (PMC10920284; doi:10.3389/fmicb.2024.1357797)

Supplementary Material

Supplementary Figure 1. Cluster Dendrogram of the Euclidean distance matrices of the microbial communities of the leaf, root, sediment, and water samples of each site. Sediment is orange, roots are brown, water is blue, and leaves are green.

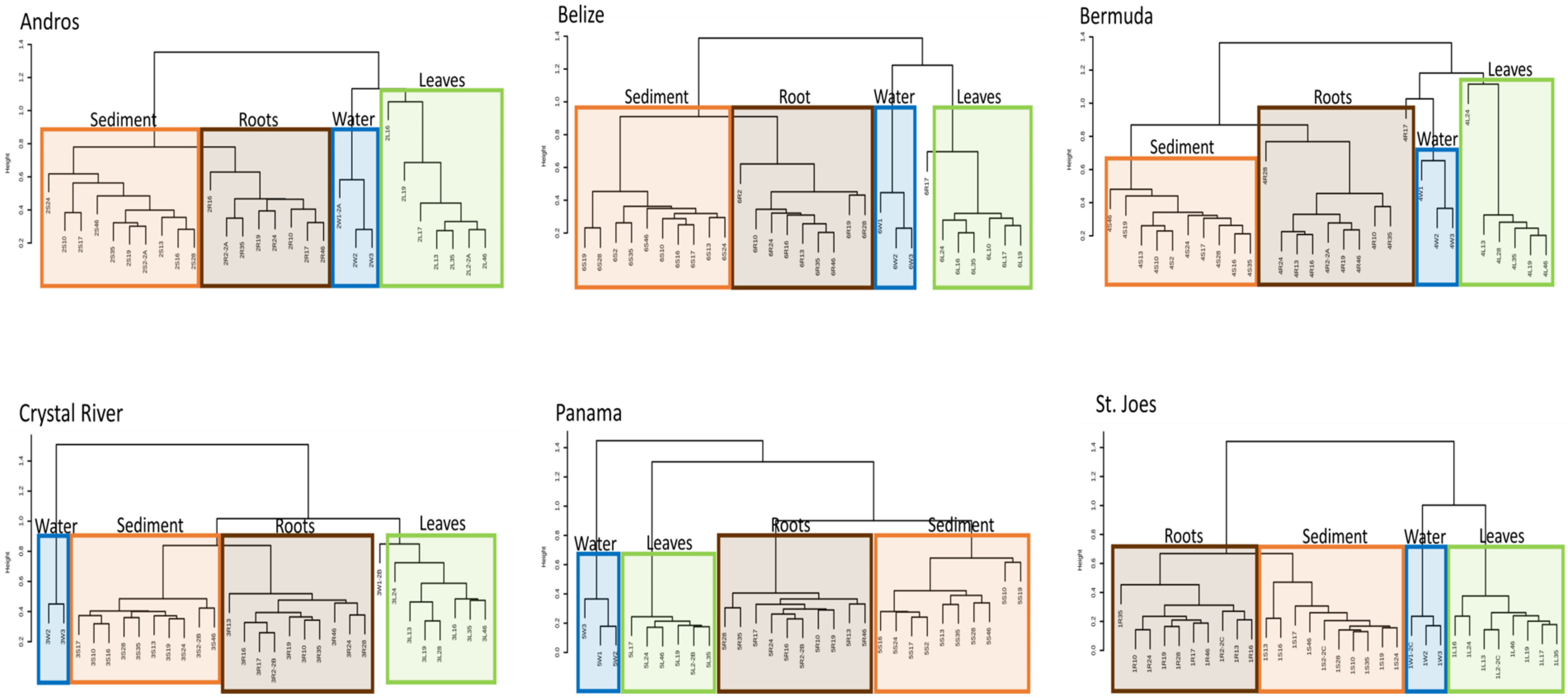

Supplement: Supplementary Figure S1 — Cluster Dendrogram of the Euclidean distance matrices of the microbial communities of the leaf, root, sediment, and water samples of each site. Sediment is orange, roots are brown, water is blue, and leaves are green. [file Image_1.PDF]
