# Supplementary material for: Microbiomes of Thalassia testudinum throughout the Atlantic Ocean, Caribbean Sea, and Gulf of Mexico are influenced by site and region while maintaining a core microbiome
Source: Front Microbiol. 2024 Feb 23;15:1357797. doi: 10.3389/fmicb.2024.1357797 (PMC10920284; doi:10.3389/fmicb.2024.1357797)
Supplement: Supplementary Table S1 — PERMANOVA of both alpha (Shannon and Chao1) and beta (Bray-Curtis and Euclidean) diversity indices to compare the microbial community composition between enriched and non-enriched plots at each site. [file Table_1.docx]

Table S1. PERMANOVA of both alpha (Shannon and Chao1) and beta (Bray-Curtis and Euclidean) diversity indices to compare the microbial community composition between enriched and non-enriched plots at each site. Bold values and asterisks indicate significant differences (p < 0.05).

|  | Leaf | | | | Root | | | |
| --- | --- | --- | --- | --- | --- | --- | --- | --- |
| Site | Shannon | Chao1 | Bray-Curtis | Euclidean | Shannon | Chao1 | Bray-Curtis | Euclidean |
| Andros | 0.042 | 0.096 | 0.055 | 0.077 | 0.241 | 0.306 | 0.798 | 0.797 |
| Belize | 0.389 | 0.555 | 0.099 | 0.136 | 0.72 | 0.717 | 0.191 | 0.168 |
| Bermuda | 0.97 | 0.971 | 0.547 | 0.818 | **0.040*** | **0.036*** | 0.055 | 0.093 |
| CrysRiver | 0.652 | 0.734 | 0.132 | 0.56 | 0.662 | 0.574 | 1 | 1 |
| Panama | 0.887 | 0.805 | 0.822 | 0.83 | 0.669 | 0.834 | 0.899 | 0.87 |
| StJoes | 0.434 | 0.692 | 0.3 | 0.528 | 0.982 | 0.384 | 0.382 | 0.146 |

Table S2. Relative abundance and taxonomic data of ASVs that compose the core microbiome of *T. testudinum* across several studies.

| ASV | Family | Genus | Study | Average rel. abundance by study | Average of avg |
| --- | --- | --- | --- | --- | --- |
| Zotu104 | Stappiaceae | Labrenzia | Present study | 0.875 |  |
|  |  |  | Rodriguez-Barreras et al. 2021 | 0.095 |  |
|  |  |  | Vogel et al. 2020 | 0.132 |  |
|  |  |  | Vogel et al. 2021a | 0.095 |  |
|  |  |  | Vogel et al. 2021b | 0.516 | 0.342771263 |
| Zotu152 | Rhodobacteraceae | NA | Present study | 0.585 |  |
|  |  |  | Rodriguez-Barreras et al. 2021 | 0.000 |  |
|  |  |  | Vogel et al. 2020 | 0.541 |  |
|  |  |  | Vogel et al. 2021a | 1.021 |  |
|  |  |  | Vogel et al. 2021b | 0.193 | 0.468090989 |
| Zotu16 | Rhodobacteraceae | Rhodovulum | Present study | 0.453 |  |
|  |  |  | Rodriguez-Barreras et al. 2021 | 0.000 |  |
|  |  |  | Vogel et al. 2020 | 0.848 |  |
|  |  |  | Vogel et al. 2021a | 0.754 |  |
|  |  |  | Vogel et al. 2021b | 1.630 | 0.737049058 |
| Zotu21 | Halieaceae | NA | Present study | 0.229 |  |
|  |  |  | Rodriguez-Barreras et al. 2021 | 0.099 |  |
|  |  |  | Vogel et al. 2020 | 0.460 |  |
|  |  |  | Vogel et al. 2021a | 1.534 |  |
|  |  |  | Vogel et al. 2021b | 1.316 | 0.727721471 |
| Zotu30 | Rhodobacteraceae | NA | Present study | 1.678 |  |
|  |  |  | Rodriguez-Barreras et al. 2021 | 0.707 |  |
|  |  |  | Vogel et al. 2020 | 0.139 |  |
|  |  |  | Vogel et al. 2021a | 1.839 |  |
|  |  |  | Vogel et al. 2021b | 0.943 | 1.061263429 |
| Zotu31 | Rhodobacteraceae | NA | Present study | 0.304 |  |
|  |  |  | Rodriguez-Barreras et al. 2021 | 0.597 |  |
|  |  |  | Vogel et al. 2020 | 0.213 |  |
|  |  |  | Vogel et al. 2021a | 1.279 |  |
|  |  |  | Vogel et al. 2021b | 0.984 | 0.675160749 |
| Zotu343 | Rhodobacteraceae | NA | Present study | 0.583 |  |
|  |  |  | Rodriguez-Barreras et al. 2021 | 0.000 |  |
|  |  |  | Vogel et al. 2020 | 0.373 |  |
|  |  |  | Vogel et al. 2021a | 0.132 |  |
|  |  |  | Vogel et al. 2021b | 0.116 | 0.240787274 |
| Zotu35 | Hyphomonadaceae | Hirschia | Present study | 3.457 |  |
|  |  |  | Rodriguez-Barreras et al. 2021 | 5.456 |  |
|  |  |  | Vogel et al. 2020 | 0.351 |  |
|  |  |  | Vogel et al. 2021a | 0.080 |  |
|  |  |  | Vogel et al. 2021b | 1.050 | 2.078845639 |
| Zotu37 | Hyphomonadaceae | NA | Present study | 0.702 |  |
|  |  |  | Rodriguez-Barreras et al. 2021 | 1.467 |  |
|  |  |  | Vogel et al. 2020 | 0.469 |  |
|  |  |  | Vogel et al. 2021a | 0.555 |  |
|  |  |  | Vogel et al. 2021b | 0.884 | 0.815542111 |
| Zotu7 | Methylophilaceae | Methylotenera | Present study | 1.912 |  |
|  |  |  | Rodriguez-Barreras et al. 2021 | 1.280 |  |
|  |  |  | Vogel et al. 2020 | 0.228 |  |
|  |  |  | Vogel et al. 2021a | 5.253 |  |
|  |  |  | Vogel et al. 2021b | 2.402 | 2.215123714 |
| Zotu78 | Rhodobacteraceae | NA | Present study | 1.421 |  |
|  |  |  | Rodriguez-Barreras et al. 2021 | 8.695 |  |
|  |  |  | Vogel et al. 2020 | 1.358 |  |
|  |  |  | Vogel et al. 2021a | 1.056 |  |
|  |  |  | Vogel et al. 2021b | 0.434 | 2.592617084 |
| Zotu8 | Rhodobacteraceae | NA | Present study | 0.865 |  |
|  |  |  | Rodriguez-Barreras et al. 2021 | 1.894 |  |
|  |  |  | Vogel et al. 2020 | 0.191 |  |
|  |  |  | Vogel et al. 2021a | 2.210 |  |
|  |  |  | Vogel et al. 2021b | 2.223 | 1.476558417 |
| Zotu91 | Hyphomonadaceae | NA | Present study | 0.775 |  |
|  |  |  | Rodriguez-Barreras et al. 2021 | 2.588 |  |
|  |  |  | Vogel et al. 2020 | 0.285 |  |
|  |  |  | Vogel et al. 2021a | 0.514 |  |
|  |  |  | Vogel et al. 2021b | 0.554 | 0.943314532 |
| Zotu99 | Rhodobacteraceae | uncultured | Present study | 1.993 |  |
|  |  |  | Rodriguez-Barreras et al. 2021 | 0.000 |  |
|  |  |  | Vogel et al. 2020 | 2.653 |  |
|  |  |  | Vogel et al. 2021a | 0.556 |  |
|  |  |  | Vogel et al. 2021b | 0.128 | 1.066130064 |

Table S3. ANOVA results of the relative abundance of the Top 20 most abundant leaf genera by site, with Bonferroni corrected p-values, followed by post-hoc Tukey’s HSD. Superscripted letters in the average relative abundance indicate groups with similar abundances. Bold indicates p < 0.05.

| Genus | p-value | corrected p-value | Site | Average relative abundance |
| --- | --- | --- | --- | --- |
| *Albimonas* | **0.0077** | 0.154 | Andros | 18.98 ± 13.68^a^ |
|  |  |  | Belize | 8.28 ± 5.32^ab^ |
|  |  |  | Bermuda | 7.55 ± 4.71^ab^ |
|  |  |  | CrystalRiver | 16.29 ± 19.20^ab^ |
|  |  |  | Panama | 2.80 ± 1.92^b^ |
|  |  |  | StJoes | 10.09 ± 2.88^ab^ |
| *Candidatus_*Endobugula | **0.0001** | **0.002** | Andros | 0.71 ± 1.11^b^ |
|  |  |  | Belize | 9.59 ± 9.40^a^ |
|  |  |  | Bermuda | 2.01 ± 2.63^b^ |
|  |  |  | CrystalRiver | 0.56 ± 1.13^b^ |
|  |  |  | Panama | 9.79 ± 4.75^a^ |
|  |  |  | StJoes | 10.19 ± 3.71^a^ |
| *Celeribacter* | **0.0002** | **0.004** | Andros | 2.07 ± 3.03^b^ |
|  |  |  | Belize | 0^b^ |
|  |  |  | Bermuda | 12.40 ± 11.78^a^ |
|  |  |  | CrystalRiver | 3.16 ± 8.94^b^ |
|  |  |  | Panama | 0^b^ |
|  |  |  | StJoes | 0.94 ± 1.12^b^ |
| *Cohaesibacter* | 0.6222 |  | Andros | 1.58 ± 4.76 |
|  |  |  | Belize | 5.15 ± 9.96 |
|  |  |  | Bermuda | 3.90 ± 5.46 |
|  |  |  | CrystalRiver | 4.22 ± 6.69 |
|  |  |  | Panama | 2.35 ± 2.95 |
|  |  |  | StJoes | 1.10 ± 2.01 |
| *Delftia* | 0.3632 |  | Andros | 3.62 ± 6.22 |
|  |  |  | Belize | 1.21 ± 2 |
|  |  |  | Bermuda | 1.59 ± 1.38 |
|  |  |  | CrystalRiver | 3.07 ± 6.32 |
|  |  |  | Panama | 1.05 ± 1.98 |
|  |  |  | StJoes | 0.21 ± 0.17 |
| *Granulosicoccus* | 0.1309 |  | Andros | 1.81 ± 2.36 |
|  |  |  | Belize | 2.12 ± 2.06 |
|  |  |  | Bermuda | 0.08 ± 0.26 |
|  |  |  | CrystalRiver | 3.41 ± 7.97 |
|  |  |  | Panama | 3.44 ± 2.54 |
|  |  |  | StJoes | 0.08 ± 0.15 |
| *Labrenzia* | 0.523 |  | Andros | 4.35 ± 9.48 |
|  |  |  | Belize | 1.76 ± 1.16 |
|  |  |  | Bermuda | 2.15 ± 2 |
|  |  |  | CrystalRiver | 0.95 ± 1.76 |
|  |  |  | Panama | 2.97 ± 2.53 |
|  |  |  | StJoes | 1.05 ± 0.88 |
| *Lentilitoribacter* | **0.0009** | **0.018** | Andros | 0^b^ |
|  |  |  | Belize | 3.54 ± 4.06^ab^ |
|  |  |  | Bermuda | 6.34 ± 4.36^a^ |
|  |  |  | CrystalRiver | 0^b^ |
|  |  |  | Panama | 4.29 ± 5.68^ab^ |
|  |  |  | StJoes | 1.90 ± 1.12^ab^ |
| *Marinagarivorans* | **0.0001** | **0.002** | Andros | 0.71 ± 1.19^b^ |
|  |  |  | Belize | 1.74 ± 2.04^b^ |
|  |  |  | Bermuda | 0.21 ± 0.60^b^ |
|  |  |  | CrystalRiver | 0.29 ± 0.81^b^ |
|  |  |  | Panama | 5.95 ± 2.53^a^ |
|  |  |  | StJoes | 1.34 ± 1.88^b^ |
| *Methylotenera* | **0.0001** | **0.002** | Andros | 0.99 ± 2.52^b^ |
|  |  |  | Belize | 7.92 ± 5.79^b^ |
|  |  |  | Bermuda | 5.11 ± 5.27^b^ |
|  |  |  | CrystalRiver | 1.14 ± 1.68^b^ |
|  |  |  | Panama | 5.70 ± 6.64^b^ |
|  |  |  | StJoes | 26.99 ± 10.97^a^ |
| *Oceanicella* | **0.0023** | **0.046** | Andros | 8.47 ± 8.58^a^ |
|  |  |  | Belize | 5.24 ± 5.05^ab^ |
|  |  |  | Bermuda | 0.94 ± 2.51^b^ |
|  |  |  | CrystalRiver | 0.82 ± 1.78^b^ |
|  |  |  | Panama | 2.68 ± 2.14^ab^ |
|  |  |  | StJoes | 1.67 ± 1.46^b^ |
| *OM27_clade* | **0.0001** | **0.002** | Andros | 0.55 ± 0.81^a^ |
|  |  |  | Belize | 1.82 ± 1.89^a^ |
|  |  |  | Bermuda | 0.32 ± 0.46^a^ |
|  |  |  | CrystalRiver | 0^a^ |
|  |  |  | Panama | 2.47 ± 2.24^a^ |
|  |  |  | StJoes | 7.90 ± 4.35^b^ |
| *Pelagibacterium* | 0.0243 | 0.486 | Andros | 4.90 ± 6.52^a^ |
|  |  |  | Belize | 0.12 ± 0.30^a^ |
|  |  |  | Bermuda | 0.02 ± 0.07^a^ |
|  |  |  | CrystalRiver | 4.17 ± 7.76^a^ |
|  |  |  | Panama | 0^a^ |
|  |  |  | StJoes | 0.92 ± 2.49^a^ |
| *Reinekea* | 0.76 |  | Andros | 3.90 ± 11.80 |
|  |  |  | Belize | 0.73 ± 1.37 |
|  |  |  | Bermuda | 0.92 ± 1.81 |
|  |  |  | CrystalRiver | 1.09 ± 2.40 |
|  |  |  | Panama | 1.28 ± 2.07 |
|  |  |  | StJoes | 2.80 ± 4.54 |
| *Rivularia_PCC-7116* | 0.0997 |  | Andros | 8.63 ± 18.25 |
|  |  |  | Belize | 1.25 ± 2.34 |
|  |  |  | Bermuda | 0.00 |
|  |  |  | CrystalRiver | 0.00 |
|  |  |  | Panama | 0.00 |
|  |  |  | StJoes | 0.00 |
| *Ruegeria* | 0.0251 | 0.502 | Andros | 0.21 ± 0.45^a^ |
|  |  |  | Belize | 1.49 ± 1.65^a^ |
|  |  |  | Bermuda | 3.70 ± 2.28^a^ |
|  |  |  | CrystalRiver | 3.47 ± 6.98^a^ |
|  |  |  | Panama | 0.12 ± 0.38^a^ |
|  |  |  | StJoes | 0.86 ± 1.07^a^ |
| *Thalassobius* | 0.007 | 0.14 | Andros | 1.29 ± 2.27^a^ |
|  |  |  | Belize | 4.09 ± 6.34^a^ |
|  |  |  | Bermuda | 0.33 ± 0.69^a^ |
|  |  |  | CrystalRiver | 0^a^ |
|  |  |  | Panama | 3.88 ± 2.53^a^ |
|  |  |  | StJoes | 0.19 ± 0.39^a^ |
| *Tropicibacter* | 0.0325 | 0.65 | Andros | 0.19 ± 0.41^a^ |
|  |  |  | Belize | 2.58 ± 6.06^a^ |
|  |  |  | Bermuda | 3.44 ± 4.01^a^ |
|  |  |  | CrystalRiver | 0^a^ |
|  |  |  | Panama | 4.50 ± 3.67^a^ |
|  |  |  | StJoes | 1.06 ± 0.87^a^ |
| *Tropicimonas* | 0.2956 |  | Andros | 0.67 ± 1.08 |
|  |  |  | Belize | 1.11 ± 1.23 |
|  |  |  | Bermuda | 2.09 ± 1.44 |
|  |  |  | CrystalRiver | 2.52 ± 4.77 |
|  |  |  | Panama | 2.73 ± 2.25 |
|  |  |  | StJoes | 1.89 ± 0.75 |
| *Vibrio* | 0.0199 | 0.398 | Andros | 4.27 ± 8.05^a^ |
|  |  |  | Belize | 0.37 ± 0.66^a^ |
|  |  |  | Bermuda | 0.08 ± 0.26^a^ |
|  |  |  | CrystalRiver | 4.60 ± 4.17^a^ |
|  |  |  | Panama | 0.49 ± 1.14^a^ |
|  |  |  | StJoes | 0.22 ± 0.22^a^ |
|  |  |  |  |  |

Table S4. ANOVA results of the relative abundance of the Top 20 most abundant root genera by site, with Bonferroni corrected p-values, followed by post-hoc Tukey’s HSD. Superscripted letters in the average relative abundance indicate groups with similar abundances. Bold indicates p < 0.05.

| *Genus* | p-value | corrected p-value | Site | Average Abundance ± Std Dev |
| --- | --- | --- | --- | --- |
| *Caldithrix* | 0.1145 |  | Andros | 2.41 ± 3.37 |
|  |  |  | Belize | 3.25 ± 3.74 |
|  |  |  | Bermuda | 2.84 ± 2.45 |
|  |  |  | CrystalRiver | 2.73 ± 0.89 |
|  |  |  | Panama | 1.64 ± 1.28 |
|  |  |  | StJoes | 5.65 ± 4.54 |
| *Candidatus_Thiodiazotropha* | **0.0008** | **0.016** | Andros | 0.65 ± 1.05^c^ |
|  |  |  | Belize | 4.53 ± 4.57^bc^ |
|  |  |  | Bermuda | 11.85 ± 9.16^abc^ |
|  |  |  | CrystalRiver | 3.53 ± 3.20^bc^ |
|  |  |  | Panama | 17.20 ± 15.81^a^ |
|  |  |  | StJoes | 14.09 ± 7.03^ab^ |
| *Delftia* | 0.2244 |  | Andros | 1.78 ± 2.95 |
|  |  |  | Belize | 0.23 ± 0.49 |
|  |  |  | Bermuda | 10.38 ± 24.36 |
|  |  |  | CrystalRiver | 0.01 ± 0.02 |
|  |  |  | Panama | 0.16 ± 0.13 |
|  |  |  | StJoes | 0.11 ± 0.12 |
| *Desulfatiglans* | **0.0194** | 0.388 | Andros | 2.71 ± 1.95^ab^ |
|  |  |  | Belize | 2.64 ± 1.50^ab^ |
|  |  |  | Bermuda | 1.50 ± 1.21^b^ |
|  |  |  | CrystalRiver | 3.98 ± 1.82^a^ |
|  |  |  | Panama | 2.28 ± 0.71^ab^ |
|  |  |  | StJoes | 2.32 ± 0.89^ab^ |
| *Desulfatitalea* | 0.0005 | **0.01** | Andros | 3.98 ± 4.36^b^ |
|  |  |  | Belize | 19.88 ± 16.65^ab^ |
|  |  |  | Bermuda | 22.21 ± 14.21^ab^ |
|  |  |  | CrystalRiver | 21.10 ± 11.98^ab^ |
|  |  |  | Panama | 34.72 ± 15.17^a^ |
|  |  |  | StJoes | 30.03 ± 11.10^a^ |
| *Desulfobulbus* | 0.0221 | 0.442 | Andros | 0.09 ± 0.24^b^ |
|  |  |  | Belize | 1.33 ± 1.75^ab^ |
|  |  |  | Bermuda | 1.60 ± 1.59^ab^ |
|  |  |  | CrystalRiver | 3.43 ± 2.36^a^ |
|  |  |  | Panama | 1.29 ± 1.38^ab^ |
|  |  |  | StJoes | 3.44 ± 4.21^a^ |
| *Desulfococcus* | 0.447 |  | Andros | 1.25 ± 0.86 |
|  |  |  | Belize | 1.89 ± 1.00 |
|  |  |  | Bermuda | 2.34 ± 2.16 |
|  |  |  | CrystalRiver | 1.69 ± 0.72 |
|  |  |  | Panama | 1.46 ± 1.17 |
|  |  |  | StJoes | 2.25 ± 1.26 |
| *Desulfomonile* | 0.0061 | 0.122 | Andros | 5.71 ± 2.98^a^ |
|  |  |  | Belize | 2.74 ± 4.47^ab^ |
|  |  |  | Bermuda | 2.20 ± 2.36^ab^ |
|  |  |  | CrystalRiver | 0.80 ± 0.57^b^ |
|  |  |  | Panama | 1.71 ± 1.08^b^ |
|  |  |  | StJoes | 1.71 ± 0.94^b^ |
| *Desulfosarcina* | 0.0385 | 0.77 | Andros | 8.54 ± 6.58 |
|  |  |  | Belize | 6.22 ± 5.03 |
|  |  |  | Bermuda | 5.81 ± 3.87 |
|  |  |  | CrystalRiver | 11.22 ± 5.67 |
|  |  |  | Panama | 3.84 ± 3.52 |
|  |  |  | StJoes | 6.24 ± 3.19 |
| *Desulfovibrio* | 0.336 |  | Andros | 5.64 ± 12.76 |
|  |  |  | Belize | 4.79 ± 8.86 |
|  |  |  | Bermuda | 1.38 ± 3.79 |
|  |  |  | CrystalRiver | 1.92 ± 3.12 |
|  |  |  | Panama | 0.19 ± 0.60 |
|  |  |  | StJoes | 0.18 ± 0.27 |
| *GWE2-31-10* | 0.0012 | **0.024** | Andros | 3.65 ± 1.75^a^ |
|  |  |  | Belize | 1.27 ± 0.92^b^ |
|  |  |  | Bermuda | 0.58 ± 0.60^b^ |
|  |  |  | CrystalRiver | 1.32 ± 0.57^b^ |
|  |  |  | Panama | 2.60 ± 2.71^ab^ |
|  |  |  | StJoes | 1.16 ± 1.35^b^ |
| *JdFR-76* | 0.1891 | 3.782 | Andros | 0.75 ± 0.71 |
|  |  |  | Belize | 1.00 ± 0.71 |
|  |  |  | Bermuda | 0.95 ± 0.81 |
|  |  |  | CrystalRiver | 1.09 ± 0.64 |
|  |  |  | Panama | 0.73 ± 0.56 |
|  |  |  | StJoes | 1.48 ± 0.42 |
| *RBG-16-49-21* | 0.0001 | **0.002** | Andros | 2.50 ± 0.93^a^ |
|  |  |  | Belize | 1.04 ± 1.00^b^ |
|  |  |  | Bermuda | 0.39 ± 0.41^b^ |
|  |  |  | CrystalRiver | 0.79 ± 0.40^b^ |
|  |  |  | Panama | 1.04 ± 0.75^b^ |
|  |  |  | StJoes | 0.56 ± 0.28^b^ |
| *Sediminispirochaeta* | 0.0348 | 0.696 | Andros | 4.68 ± 5.40 |
|  |  |  | Belize | 1.68 ± 1.88 |
|  |  |  | Bermuda | 0.98 ± 1.07 |
|  |  |  | CrystalRiver | 2.54 ± 3.16 |
|  |  |  | Panama | 0.29 ± 0.42 |
|  |  |  | StJoes | 1.94 ± 2.61 |
| *SEEP-SRB1* | 0.0001 | **0.002** | Andros | 1.69 ± 1.24^b^ |
|  |  |  | Belize | 2.49 ± 1.55^b^ |
|  |  |  | Bermuda | 1.81 ± 1.86^b^ |
|  |  |  | CrystalRiver | 5.82 ± 2.58^a^ |
|  |  |  | Panama | 1.90 ± 1.32^b^ |
|  |  |  | StJoes | 2.85 ± 1.23^b^ |
| *Spirochaeta_2* | 0.143 |  | Andros | 7.49 ± 4.15 |
|  |  |  | Belize | 4.83 ± 2.72 |
|  |  |  | Bermuda | 4.89 ± 7.42 |
|  |  |  | CrystalRiver | 8.78 ± 4.36 |
|  |  |  | Panama | 9.68 ± 5.38 |
|  |  |  | StJoes | 5.52 ± 3.25 |
| *Subgroup_23* | 0.0001 | **0.002** | Andros | 4.01 ± 2.79^a^ |
|  |  |  | Belize | 0.42 ± 0.25^b^ |
|  |  |  | Bermuda | 0.57 ± 0.80^b^ |
|  |  |  | CrystalRiver | 0.70 ± 0.36^b^ |
|  |  |  | Panama | 0.41 ± 0.42^b^ |
|  |  |  | StJoes | 0.86 ± 0.36^b^ |
| *Sulfurimonas* | 0.3793 |  | Andros | 0.08 ± 0.08 |
|  |  |  | Belize | 0.76 ± 1.56 |
|  |  |  | Bermuda | 0.13 ± 0.24 |
|  |  |  | CrystalRiver | 6.05 ± 16.49 |
|  |  |  | Panama | 0.61 ± 1.74 |
|  |  |  | StJoes | 0.69 ± 1.31 |
| *Sva0081_sediment_group* | 0.0001 | **0.002** | Andros | 1.03 ± 1.70^b^ |
|  |  |  | Belize | 1.96 ± 1.36^b^ |
|  |  |  | Bermuda | 2.28 ± 2.28^b^ |
|  |  |  | CrystalRiver | 5.73 ± 3.60^a^ |
|  |  |  | Panama | 0.65 ± 0.48^b^ |
|  |  |  | StJoes | 2.01 ± 1.12^b^ |
| *Thalassospira* | 0.483 |  | Andros | 0 |
|  |  |  | Belize | 5.85 ± 18.30 |
|  |  |  | Bermuda | 0 |
|  |  |  | CrystalRiver | 0 |
|  |  |  | Panama | 0 |
|  |  |  | StJoes | 0.01 ± 0.01 |

Table S5. ANOVA results of the relative abundance of the Top 20 most abundant sediment genera by site, with Bonferroni corrected p-values, followed by post-hoc Tukey’s HSD. Superscripted letters in the average relative abundance indicate groups with similar abundances. Bold indicates p < 0.05.

| *Genus* | p-value | corrected p-value | Site | Average Abundance ± std dev |
| --- | --- | --- | --- | --- |
| *Candidatus_Thiobios* | 0.0001 | **0.002** | Andros | 0.24 ± 0.34^c^ |
|  |  |  | Belize | 0.36 ± 0.21^c^ |
|  |  |  | Bermuda | 0.54 ± 0.41^c^ |
|  |  |  | CrystalRiver | 1.89 ± 1.31^b^ |
|  |  |  | Panama | 0.38 ± 0.27^c^ |
|  |  |  | StJoes | 3.46 ± 1.42^a^ |
| *Coxiella* | 0.0001 | **0.002** | Andros | 14.20 ± 7.90^a^ |
|  |  |  | Belize | 3.40 ± 1.85^b^ |
|  |  |  | Bermuda | 1.96 ± 1.27^b^ |
|  |  |  | CrystalRiver | 3.81 ± 0.75^b^ |
|  |  |  | Panama | 2.79 ± 1.10^b^ |
|  |  |  | StJoes | 0.14 ± 0.09^b^ |
| *Delftia* | 0.0001 | **0.002** | Andros | 8.88 ± 3.28^b^ |
|  |  |  | Belize | 17.78 ± 4.88^a^ |
|  |  |  | Bermuda | 8.26 ± 4.56^b^ |
|  |  |  | CrystalRiver | 2.20 ± 0.49^c^ |
|  |  |  | Panama | 9.16 ± 2.64^b^ |
|  |  |  | StJoes | 0.04 ± 0.03^c^ |
| *Desulfatiglans* | 0.0001 | **0.002** | Andros | 1.64 ± 1.05^c^ |
|  |  |  | Belize | 4.44 ± 1.50^b^ |
|  |  |  | Bermuda | 1.61 ± 0.82^c^ |
|  |  |  | CrystalRiver | 9.07 ± 2.86^a^ |
|  |  |  | Panama | 3.19 ± 1.32^bc^ |
|  |  |  | StJoes | 2.46 ± 1.34^bc^ |
| *Desulfatitalea* | 0.0001 | **0.002** | Andros | 1.00 ± 0.77 |
|  |  |  | Belize | 2.28 ± 1.14 |
|  |  |  | Bermuda | 1.74 ± 0.92 |
|  |  |  | CrystalRiver | 0.42 ± 0.24 |
|  |  |  | Panama | 0.57 ± 0.51 |
|  |  |  | StJoes | 0.85 ± 1.23 |
| *Desulfococcus* | 0.0001 | **0.002** | Andros | 0.93 ± 0.49^b^ |
|  |  |  | Belize | 1.07 ± 0.51^b^ |
|  |  |  | Bermuda | 0.66 ± 0.23^b^ |
|  |  |  | CrystalRiver | 0.95 ± 0.65^b^ |
|  |  |  | Panama | 0.54 ± 0.51^b^ |
|  |  |  | StJoes | 2.62 ± 0.68^a^ |
| *Desulfosarcina* | 0.0001 | **0.002** | Andros | 0.39 ± 0.18^b^ |
|  |  |  | Belize | 0.45 ± 0.32^b^ |
|  |  |  | Bermuda | 0.77 ± 0.44^b^ |
|  |  |  | CrystalRiver | 1.48 ± 1.29^b^ |
|  |  |  | Panama | 0.81 ± 0.73^b^ |
|  |  |  | StJoes | 5.75 ± 2.60^a^ |
| *Herbaspirillum* | 0.0001 | **0.002** | Andros | 1.32 ± 0.88^ab^ |
|  |  |  | Belize | 0.74 ± 0.36^b^ |
|  |  |  | Bermuda | 1.02 ± 0.25^b^ |
|  |  |  | CrystalRiver | 0.85 ± 0.60^b^ |
|  |  |  | Panama | 1.74 ± 0.53^a^ |
|  |  |  | StJoes | 0.01 ± 0.01^c^ |
| *Pir4_lineage* | 0.0001 | **0.002** | Andros | 0.77 ± 0.65^b^ |
|  |  |  | Belize | 3.54 ± 3.18^a^ |
|  |  |  | Bermuda | 0.19 ± 0.12^b^ |
|  |  |  | CrystalRiver | 0.96 ± 0.29^b^ |
|  |  |  | Panama | 0.83 ± 0.37^b^ |
|  |  |  | StJoes | 1.40 ± 0.41^b^ |
| *Pirellula* | 0.0001 | **0.002** | Andros | 0.39 ± 0.20^bc^ |
|  |  |  | Belize | 0.65 ± 0.36^b^ |
|  |  |  | Bermuda | 0.30 ± 0.11^bc^ |
|  |  |  | CrystalRiver | 0.23 ± 0.18c |
|  |  |  | Panama | 0.39 ± 0.19^bc^ |
|  |  |  | StJoes | 3.00 ± 0.50^a^ |
| *Sediminispirochaeta* | 0.0236 |  | Andros | 4.09 ± 3.16^a^ |
|  |  |  | Belize | 1.40 ± 0.76^b^ |
|  |  |  | Bermuda | 1.96 ± 1.62^bc^ |
|  |  |  | CrystalRiver | 1.84 ± 0.35^ab^ |
|  |  |  | Panama | 2.56 ± 1.54^ab^ |
|  |  |  | StJoes | 1.34 ± 1.36^b^ |
| *SEEP-SRB1* | 0.0001 | **0.002** | Andros | 3.60 ± 1.98^c^ |
|  |  |  | Belize | 7.14 ± 2.05^b^ |
|  |  |  | Bermuda | 4.78 ± 1.62^bc^ |
|  |  |  | CrystalRiver | 10.23 ± 2.06^a^ |
|  |  |  | Panama | 5.13 ± 1.63^bc^ |
|  |  |  | StJoes | 4.83 ± 2.00^bc^ |
| *Spirochaeta_2* | 0.0001 | **0.002** | Andros | 14.54 ± 5.93^b^ |
|  |  |  | Belize | 9.28 ±2.15^c^ |
|  |  |  | Bermuda | 6.22 ± 1.38^cd^ |
|  |  |  | CrystalRiver | 26.35 ± 6.27^a^ |
|  |  |  | Panama | 7.15 ± 2.85^cd^ |
|  |  |  | StJoes | 2.78 ± 0.60^d^ |
| *Subgroup_10* | 0.114 |  | Andros | 4.56 ± 2.82 |
|  |  |  | Belize | 3.39 ± 2.41 |
|  |  |  | Bermuda | 4.75 ± 3.89 |
|  |  |  | CrystalRiver | 2.46 ± 1.48 |
|  |  |  | Panama | 6.14 ± 5.24 |
|  |  |  | StJoes | 2.29 ± 2.22 |
| *Subgroup_23* | 0.0001 | **0.002** | Andros | 2.28 ± 0.913^b^ |
|  |  |  | Belize | 2.06 ± 0.81^b^ |
|  |  |  | Bermuda | 6.09 ± 2.55^a^ |
|  |  |  | CrystalRiver | 2.70 ± 2.64^b^ |
|  |  |  | Panama | 2.42 ± 1.43^b^ |
|  |  |  | StJoes | 1.50 ± 0.52^b^ |
| *Sulfurovum* | 0.0004 | **0.008** | Andros | 0.88 ± 1.27^c^ |
|  |  |  | Belize | 6.22 ± 5.51^abc^ |
|  |  |  | Bermuda | 20.34 ± 14.78^a^ |
|  |  |  | CrystalRiver | 4.57 ± 6.13^bc^ |
|  |  |  | Panama | 17.37 ± 18.62^ab^ |
|  |  |  | StJoes | 2.66 ± 2.85^c^ |
| *Sva0081_sediment_group* | 0.0001 | **0.002** | Andros | 6.41 ± 2.54^b^ |
|  |  |  | Belize | 5.49 ± 1.66^b^ |
|  |  |  | Bermuda | 5.03 ± 2.09^b^ |
|  |  |  | CrystalRiver | 8.21 ± 1.51^b^ |
|  |  |  | Panama | 8.17 ± 3.42^b^ |
|  |  |  | StJoes | 19.18 ± 5.64^a^ |
| *Thiogranum* | 0.0001 | **0.002** | Andros | 0.62 ± 0.57^bc^ |
|  |  |  | Belize | 0.42 ± 0.36^c^ |
|  |  |  | Bermuda | 0.67 ± 0.35^bc^ |
|  |  |  | CrystalRiver | 0.46 ± 0.30^c^ |
|  |  |  | Panama | 1.78 ± 1.41^a^ |
|  |  |  | StJoes | 1.59 ± 0.55^ab^ |
| *Thiohalophilus* | 0.0001 | **0.002** | Andros | 0.01 ± 0.03^b^ |
|  |  |  | Belize | 0.03 ± 0.06^b^ |
|  |  |  | Bermuda | 0.36 ± 0.25^b^ |
|  |  |  | CrystalRiver | 0^b^ |
|  |  |  | Panama | 3.66 ± 1.41^a^ |
|  |  |  | StJoes | 0.26 ± 0.29^b^ |
| *Woeseia* | 0.0014 | **0.028** | Andros | 0.91 ± 0.74^b^ |
|  |  |  | Belize | 0.44 ± 0.32^b^ |
|  |  |  | Bermuda | 2.62 ± 1.59^ab^ |
|  |  |  | CrystalRiver | 0.56 ± 0.23^b^ |
|  |  |  | Panama | 0.74 ± 0.37^b^ |
|  |  |  | StJoes | 3.45 ± 4.07^a^ |

Table S6. ANOVA results of the relative abundance of the Top 20 most abundant water genera by site, with Bonferroni corrected p-values, followed by post-hoc Tukey’s HSD. Superscripted letters in the average relative abundance indicate groups with similar abundances. Bold indicates p < 0.05.

| *Genus* | p-value | corrected p-value | Site | Average Abundance |
| --- | --- | --- | --- | --- |
| *Bacillus* | 0.2135 |  | Andros | 3.72 ± 0.63 |
|  |  |  | Belize | 2.06 ± 2.00 |
|  |  |  | Bermuda | 1.73 ± 1.88 |
|  |  |  | CrystalRiver | 3.67 ± 3.18 |
|  |  |  | Panama | 0.61 ± 1.05 |
|  |  |  | StJoes | 0.87 ± 0.35 |
| *Candidatus_Actinomarina* | 0.0176 | 0.352 | Andros | 0^b^ |
|  |  |  | Belize | 1.24 ± 1.42^ab^ |
|  |  |  | Bermuda | 2.06 ± 2.36^ab^ |
|  |  |  | CrystalRiver | 0.29 ± 0.26^b^ |
|  |  |  | Panama | 5.14 ± 2.54^a^ |
|  |  |  | StJoes | 1.61 ± 0.31^ab^ |
| *Clade_Ia* | 0.0004 | **0.008** | Andros | 0^b^ |
|  |  |  | Belize | 0.17 ± 0.16^b^ |
|  |  |  | Bermuda | 2.17 ± 0.97^a^ |
|  |  |  | CrystalRiver | 0.17 ± 0.16^b^ |
|  |  |  | Panama | 1.46 ± 0.56^a^ |
|  |  |  | StJoes | 1.12 ± 0.05^ab^ |
| *Corynebacterium_1* | 0.6677 |  | Andros | 2.71 ± 0.09 |
|  |  |  | Belize | 1.74 ± 1.61 |
|  |  |  | Bermuda | 2.63 ± 2.26 |
|  |  |  | CrystalRiver | 1.94 ± 1.89 |
|  |  |  | Panama | 0.87 ± 0.94 |
|  |  |  | StJoes | 1.41 ± 1.36 |
| *Delftia* | 0.0274 | 0.548 | Andros | 31.44 ± 3.20^ab^ |
|  |  |  | Belize | 49.47 ± 10.23^a^ |
|  |  |  | Bermuda | 34.07 ± 19.39^ab^ |
|  |  |  | CrystalRiver | 18.30 ± 8.18^b^ |
|  |  |  | Panama | 42.82 ± 7.27^ab^ |
|  |  |  | StJoes | 23.66 ± 3.05^ab^ |
| *Dolosigranulum* | 0.7897 |  | Andros | 0.75 ± 1.30 |
|  |  |  | Belize | 0.86 ± 0.92 |
|  |  |  | Bermuda | 1.39 ± 1.35 |
|  |  |  | CrystalRiver | 0.66 ± 0.57 |
|  |  |  | Panama | 0.58 ± 0.35 |
|  |  |  | StJoes | 0.33 ± 0.30 |
| *Escherichia/Shigella* | 0.0098 | 0.196 | Andros | 3.16 ± 1.29^a^ |
|  |  |  | Belize | 0.97 ± 0.51^b^ |
|  |  |  | Bermuda | 1.60 ± 0.29^ab^ |
|  |  |  | CrystalRiver | 1.07 ± 0.49^b^ |
|  |  |  | Panama | 0.97 ± 0.61^b^ |
|  |  |  | StJoes | 0.84 ± 0.38^b^ |
| *Herbaspirillum* | 0.1758 |  | Andros | 8.07 ± 2.28 |
|  |  |  | Belize | 7.22 ± 4.88 |
|  |  |  | Bermuda | 7.88 ± 3.14 |
|  |  |  | CrystalRiver | 8.21 ± 2.19 |
|  |  |  | Panama | 9.74 ± 3.20 |
|  |  |  | StJoes | 2.77 ± 0.73 |
| *HIMB11* | 0.0003 | **0.006** | Andros | 0.47 ± 0.31^c^ |
|  |  |  | Belize | 1.30 ± 2.04^c^ |
|  |  |  | Bermuda | 1.11 ± 1.00^c^ |
|  |  |  | CrystalRiver | 11.12 ± 4.84^a^ |
|  |  |  | Panama | 3.61 ± 1.56^bc^ |
|  |  |  | StJoes | 9.06 ± 0.93^ab^ |
| *Leucothrix* | 0.3623 |  | Andros | 3.80 ± 1.89 |
|  |  |  | Belize | 1.75 ± 1.52 |
|  |  |  | Bermuda | 3.35 ± 3.93 |
|  |  |  | CrystalRiver | 1.93 ± 1.68 |
|  |  |  | Panama | 0.40 ± 0.70 |
|  |  |  | StJoes | 1.20 ± 0.21 |
| *Lewinella* | 0.1892 |  | Andros | 1.52 ± 0.12 |
|  |  |  | Belize | 0.55 ± 0.91 |
|  |  |  | Bermuda | 1.69 ± 1.48 |
|  |  |  | CrystalRiver | 0.94 ± 0.87 |
|  |  |  | Panama | 0.10 ± 0.17 |
|  |  |  | StJoes | 0.54 ± 0.11 |
| *Litoricola* | 0.0014 | **0.028** | Andros | 2.42 ± 1.65^bc^ |
|  |  |  | Belize | 1.22 ± 0.73^bc^ |
|  |  |  | Bermuda | 0.49 ± 0.12^c^ |
|  |  |  | CrystalRiver | 6.07 ± 3.90^ab^ |
|  |  |  | Panama | 0.56 ± 0.81^c^ |
|  |  |  | StJoes | 7.81 ± 1.41^a^ |
| *Marinobacterium* | 0.0001 | **0.002** | Andros | 0^b^ |
|  |  |  | Belize | 0.37 ± 0.22^b^ |
|  |  |  | Bermuda | 0^b^ |
|  |  |  | CrystalRiver | 0.07 ± 0.12^b^ |
|  |  |  | Panama | 4.24 ± 1.51^a^ |
|  |  |  | StJoes | 0^b^ |
| *NS5_marine_group* | 0.0013 | **0.026** | Andros | 1.39 ± 1.23^b^ |
|  |  |  | Belize | 1.60 ± 1.16^b^ |
|  |  |  | Bermuda | 3.03 ± 2.95^b^ |
|  |  |  | CrystalRiver | 16.97 ± 8.07^a^ |
|  |  |  | Panama | 2.29 ± 0.90^b^ |
|  |  |  | StJoes | 7.68 ± 0.52^ab^ |
| *Pseudomonas* | 0.5661 |  | Andros | 1.45 ± 0.98 |
|  |  |  | Belize | 1.11 ± 1.14 |
|  |  |  | Bermuda | 1.66 ± 1.81 |
|  |  |  | CrystalRiver | 0.86 ± 1.19 |
|  |  |  | Panama | 0.29 ± 0.51 |
|  |  |  | StJoes | 0.31 ± 0.15 |
| *Shewanella* | 0.0772 |  | Andros | 2.27 ± 0.89 |
|  |  |  | Belize | 0.10 ± 0.96 |
|  |  |  | Bermuda | 1.56 ± 1.55 |
|  |  |  | CrystalRiver | 0.85 ± 0.83 |
|  |  |  | Panama | 0.03 ± 0.05 |
|  |  |  | StJoes | 0.19 ± 0.08 |
| *Staphylococcus* | 0.751 |  | Andros | 1.22 ± 1.35 |
|  |  |  | Belize | 0.92 ± 0.80 |
|  |  |  | Bermuda | 1.57 ± 1.22 |
|  |  |  | CrystalRiver | 0.96 ± 0.62 |
|  |  |  | Panama | 0.58 ± 0.78 |
|  |  |  | StJoes | 0.58 ± 0.18 |
| *SUP05_cluster* | 0.0001 | **0.002** | Andros | 0^b^ |
|  |  |  | Belize | 0^b^ |
|  |  |  | Bermuda | 0^b^ |
|  |  |  | CrystalRiver | 0.19 ± 0.19^b^ |
|  |  |  | Panama | 0^b^ |
|  |  |  | StJoes | 11.12 ± 1.43^a^ |
| *Synechococcus_CC9902* | 0.0001 | **0.002** | Andros | 0.48 ± 0.24^bc^ |
|  |  |  | Belize | 1.26 ± 1.48^bc^ |
|  |  |  | Bermuda | 2.60 ± 0.48^b^ |
|  |  |  | CrystalRiver | 0.03 ± 0.05^c^ |
|  |  |  | Panama | 8.94 ± 0.92^a^ |
|  |  |  | StJoes | 0.73 ± 0.60^bc^ |
| *Vibrio* | 0.188 |  | Andros | 1.02 ± 0.77 |
|  |  |  | Belize | 0.77 ± 0.85 |
|  |  |  | Bermuda | 1.83 ± 1.35 |
|  |  |  | CrystalRiver | 1.34 ± 0.15 |
|  |  |  | Panama | 0.21 ± 0.21 |
|  |  |  | StJoes | 0.61 ± 0.24 |

Table S7. Top 20 most abundant genera by sample type with a list of possible functions and/or interesting facts relating to seagrasses, marine flora or fauna, or other plant systems.

| Leaf Top 20 Genera | Notes |
| --- | --- |
| *Albimonas* | found in pacific |
| *Candidatus endobugula* | Associated with producing bryostatins in bryozans which have been shown to chemically deter fish from eating their larva (Davidson *et al.* 2001; Lim and Haygood 2004; Lopanik, Lindquist and Targett 2004) |
| *Celeribacter* | Some species contain genes associated with import and export of C, N, P, S (Wang *et al.* 2020) |
| *Cohaesibacter* | associated with stony coral tissue loss disease (Rosales *et al.* 2020) |
| *Delftia* | Promotes plant growth, antagonizes pathogens, fixes N in lupine plants (Agafonova *et al.* 2017) |
| *Granulosicoccus* | Associated with black/purple tissue in Z. muelleri (Hurtado-McCormick *et al.* 2020) and associated with seagrasses (Kurilenko *et al.* 2010) |
| *Labrenzia* | some contain antimicrobial activity against bacteria and fungi (Amiri Moghaddam *et al.* 2018; Raj Sharma *et al.* 2019); also forms biofilms (Zaynab *et al.* 2021) |
| *Lentilitoribacter* | relevance not found |
| *Marinagarivorans* | isolated from marine algae; can degraded agar (Guo *et al.* 2016) |
| *Mathylotenera* | Denitrifyiers (Ildar *et al.* 2013) |
| *Oceanicella* | relevance not found |
| *OM27_clade* | putative predatory bacteria (Orsi *et al.* 2016) |
| *Pelagibacterium* | relevance not found |
| *Reinekea* | some species can degrade marine algae (Hakamada, Ohkubo and Ohashi 2014) |
| *Rivularia_PCC-7116* | cyanobacteria (Tomáš *et al.* 2022) |
| *Ruegeria* | possible probiotics in corals due to association with coral pathogens (Kitamura *et al.* 2021); algicidal properties (Riclea *et al.* 2012); associated with seagrasses before (Weidner *et al.* 2000) |
| *Thalassobius* | associated with lobster epizootic shell disease (Quinn *et al.* 2012) |
| *Tropicibacter* | some species can degrade phthalate (Iwaki, Nishimura and Hasegawa 2012) |
| *Tropicimonas* | isolated from clam sediments (Shin *et al.* 2012) |
| *Vibrio* | can be associated with diseases and known to be reduced in waters of seagrass beds (Reusch *et al.* 2021) |
|  |  |
| Root Top 20 Genera | Notes |
| *Caldithrix* | Aanaerobic, denitrifier (Miroshnichenko *et al.* 2003) |
| *Candidtus thiodiazotropha* | sulfur oxidizing, nitogen fixing in lucinid clams (Petersen *et al.* 2016) |
| *Delftia* | Promotes plant growth, antagonizes pathogens, fixes N in lupine plants (Agafonova *et al.* 2017) |
| *Desulfatiglans* | sulfate reducers (Galushko and Kuever 2019) |
| *Desulfatitalea* | sulfate reducer (Fishman *et al.* 2013) |
| *Desulfobulbus* | sulfate reducer (Higashioka *et al.* 2013) |
| *Desulfococcus* | sulfate reducers (Bridge, White and Gadd 1999) |
| *Desulfomonile* | sulphate reducers associated with stressed seagrass roots (Halophila ovalis; Martin *et al.* 2020) |
| *Desulfosarcina* | sulfate reducers (Kleindienst *et al.* 2014) |
| *Desulfovibrio* | sulfate reducers (Heidelberg *et al.* 2004) |
| *GWE2-31-10* | relevance not found |
| *JdFR-76* | associated with disease root rot (Ganoderma) disease suppresion (Goh *et al.* 2020) |
| *RBG-16-49-21* | relevance not found |
| *Sediminispirochaeta* | relevance not found |
| *SEEP-SRB1* | associate with stressed seagrass roots (H. ovalis; Martin *et al.* 2020) |
| *Spirochaeta_2* | sulfur oxidizers (Dubinina *et al.* 2011) |
| *Subgroups_23* | relevance not found |
| *Sulfurimonas* | sulfur oxidizers associated with seagrass rhizospheres (Enhaulus acoroides; Zhang *et al.* 2022) |
| *Sva0081_sediment group* | deltaproteobacteria, likely involved in sulfur cycling (Mußmann *et al.* 2005) |
| *Thalassospira* | relevance not found |
|  |  |
| Sediment Top 20 Genera | Notes |
| *Candidatus thiobios* | sulfur oxidizer associated with giant marine ciliate (Rinke et al. 2009) |
| *Coxiella* | some species are known as pathogens of marine mammals (Kersh et al. 2012) |
| *Delftia* | Promotes plant growth, antagonizes pathogens, fixes N in lupine plants (Agafonova et al. 2017) |
| *Desulfatiglans* | sulfate reducers (Galushko and Kuever 2019) |
| *Desulfatitalea* | sulfate reducer (Fishman et al. 2013) |
| *Desulfoccocus* | sulfate reducers (Bridge, White and Gadd 1999) |
| *Desulfosarcina* | sulfate reducers (Kleindienst et al. 2014) |
| *Herbaspirillum* | contains N fixers (Ureta et al. 1995) |
| *Pir4_lineage* | relevance not found |
| *Pirellula* | relevance not found |
| *Sediminispirochaeta* | relevance not found |
| *SEEP-SRB1* | associate with stressed seagrass roots (H. ovalis; Martin et al. 2020) |
| *Spirochaeta_2* | sulfur oxidizers (Dubinina et al. 2011) |
| *Subgroup_10* | relevance not found |
| *Subgroup_23* | relevance not found |
| *Sulfurovum* | some known to fix CO2 and also oxidize sulfur (Jeon et al. 2017) |
| *Sva0081_sedimet group* | deltaproteobacteria, likely involved in sulfur cycling (Mußmann et al. 2005) |
| *Thiogranum* | sulfur oxidizer associated with mangrove sediments (Li et al. 2019) |
| *Thiohalophilus* | sulfur oxidizer associated with mangrove sediment (Li et al. 2022) |
| *Woeseia* | associated with marine biofilms (Zhang et al. 2019); associated with roots of H. ovalis in higher levels of Fe and As (Martin et al. 2022) |
|  |  |
| Water Top 20 Genera | Notes |
| *Bacillus* | associated with epiphitic communities on seagrasses (Mishra and Mohanraju 2018) |
| *Candidatus Actinomarina* | associated with harmful algal blooms in estuary water (Fortin et al. 2022) |
| *Clade Ia* | relevance not found |
| *Corynebacterium 1* | seagrass can reduce this in the water potential pathogen of people (Lamb et al. 2017) |
| *Delftia* | Promotes plant growth, antagonizes pathogens, fixes N in lupine plants (Agafonova et al. 2017) |
| *Dolosigranulum* | associated with human nasal passages (Lécuyer et al. 2007) |
| *Escherichia/Shigella* | common pathogens |
| *Herbaspirillum* | contains N fixers (Ureta et al. 1995) |
| *HIMB11* | planktonic marine bacterium (Durham et al. 2014) |
| *Leucothrix* | sulfur oxidizers (Brock 2006) |
| *Lewinella* | associated with roots of H. ovalis in higher levels of Fe and As (Martin et al. 2022) |
| *Litoricola* | relevance not found |
| *Marinobacterium* | associated with low-tide in mangroves (Becker et al. 2020), marine nitrogen fixers (Alfaro-Espinoza and Ullrich 2014) |
| *NS5 marine group* | associated with eutrophication and correlates with silicate (Kopprio et al. 2021) |
| *Pseudomonas* | nitrate and phosphate positively correlate with pseudomonas in T. hemprichii roots and nitrite in leaves (Cai et al. 2021) |
| *Shewanella* | considered a growth limiting bacteria for Chattonella blooms and is found on the biofilm and water of Z. marina (Inaba et al. 2019) |
| *Staphylococcus* | isolated from seagrasses before and shown to have antifungal activity (Bibi et al. 2018) |
| *SUP05-cluster* | associated with water column of mangroves (Laas et al. 2022) |
| *Synechococcus_CC9902* | indicator of eutrophication (Kopprio 2021), sulfur oxidizer that can produce nitrite (Shah, Chang and Morris 2017) |
| *Vibrio* | can be associated with diseases and known to be reduced in waters of seagrass beds (Reusch et al. 2021) |

References for Table S7.

Agafonova N V, Doronina N V, Kaparullina EN *et al.* A novel *Delftia* plant symbiont capable of autotrophic methylotrophy. *Microbiology* 2017;**86**:96–105.

Alfaro-Espinoza G, Ullrich MS. Marinobacterium mangrovicola sp. nov., a marine nitrogen-fixing bacterium isolated from mangrove roots of Rhizophora mangle. *Int J Syst Evol Microbiol* 2014;**64**:3988–93.

Amiri Moghaddam J, Dávila-Céspedes A, Kehraus S *et al.* Cyclopropane-Containing Fatty Acids from the Marine Bacterium *Labrenzia* sp. 011 with Antimicrobial and GPR84 Activity. *Mar Drugs* 2018;**16**, DOI: 10.3390/md16100369.

Becker C, Weber L, Suca J *et al.* Microbial and nutrient dynamics in mangrove, reef, and seagrass waters over tidal and diurnal time scales. *Aquat Microb Ecol* 2020;**85**:101–19.

Bibi F, Naseer MI, Hassan AM *et al.* Diversity and antagonistic potential of bacteria isolated from marine grass *Halodule uninervis*. *3 Biotech* 2018;**8**:48.

Bridge TAM, White C, Gadd GM. Extracellular metal-binding activity of the sulphate-reducing bacterium *Desulfococcus multivorans*. *Microbiology* 1999;**145**.

Brock TD. The Genus *Leucothrix* BT - The Prokaryotes: A Handbook on the Biology of Bacteria Volume 6: Proteobacteria: Gamma Subclass. In: Dworkin M, Falkow S, Rosenberg E, et al. (eds.). New York, NY: Springer New York, 2006, 931–8.

Cai Z, Zhou L, Liu L *et al.* Bacterial epiphyte and endophyte communities of seagrass Thalassia hemprichii: the impact of feed extract solution. *Environ Microbiol Rep* 2021;**13**:757–72.

Davidson SK, Allen SW, Lim GE *et al.* Evidence for the Biosynthesis of Bryostatins by the Bacterial Symbiont “*Candidatus* Endobugula sertula” of the Bryozoan *Bugula neritina*. *Appl Environ Microbiol* 2001;**67**:4531–7.

Dubinina G, Grabovich M, Leshcheva N *et al.* *Spirochaeta perfilievii* sp. nov., an oxygen-tolerant, sulfide-oxidizing, sulfur- and thiosulfate-reducing spirochaete isolated from a saline spring. *Int J Syst Evol Microbiol* 2011;**61**.

Durham BP, Grote J, Whittaker KA *et al.* Draft genome sequence of marine alphaproteobacterial strain HIMB11, the first cultivated representative of a unique lineage within the Roseobacter clade possessing an unusually small genome. *Stand Genomic Sci* 2014;**9**:632–45.

Fishman KS, Akimov VN, Suzina NE *et al.* Sulfate-reducing bacteria Desulfobulbus sp. strain BH from a freshwater lake in Guizhou Province, China. *Inl Water Biol* 2013;**6**:13–7.

Fortin SG, Song B, Anderson IC *et al.* Blooms of the harmful algae Margalefidinium polykrikoides and Alexandrium monilatum alter the York River Estuary microbiome. *Harmful Algae* 2022;**114**:102216.

Galushko A, Kuever J. *Desulfatiglans*. *Bergey’s Man Syst Archaea Bact* 2019:1–4.

Goh YK, Zoqratt MZ, Goh YK *et al.* Determining Soil Microbial Communities and Their Influence on *Ganoderma* Disease Incidences in Oil Palm (*Elaeis guineensis*) via High-Throughput Sequencing. *Biology (Basel)* 2020;**9**, DOI: 10.3390/biology9120424.

Guo LY, Li DQ, Sang J *et al.* Marinagarivorans algicola gen. nov., sp nov., isolated from marine algae. *Int J Syst Evol Microbiol* 2016;**66**:1593–9.

Hakamada Y, Ohkubo Y, Ohashi S. Purification and characterization of β-Mannanase from Reinekea sp. KIT-YO10 with transglycosylation activity. *Biosci Biotechnol Biochem* 2014;**78**:722–8.

Heidelberg JF, Seshadri R, Haveman SA *et al.* The genome sequence of the anaerobic, sulfate-reducing bacterium Desulfovibrio vulgaris Hildenborough. *Nat Biotechnol* 2004;**22**:554–9.

Higashioka Y, Kojima H, Watanabe M *et al.* *Desulfatitalea tepidiphila* gen. nov., sp. nov., a sulfate-reducing bacterium isolated from tidal flat sediment. *Int J Syst Evol Microbiol* 2013;**63**.

Hurtado-McCormick V, Kahlke T, Krix D *et al.* Seagrass leaf reddening alters the microbiome of *Zostera muelleri*. *Mar Ecol Prog Ser* 2020;**646**:29–44.

Ildar M, G. KM, E. LM *et al.* Insights into Denitrification in Methylotenera mobilis from Denitrification Pathway and Methanol Metabolism Mutants. *J Bacteriol* 2013;**195**:2207–11.

Inaba N, Trainer VL, Nagai S *et al.* Dynamics of seagrass bed microbial communities in artificial Chattonella blooms: A laboratory microcosm study. *Harmful Algae* 2019;**84**:139–50.

Iwaki H, Nishimura A, Hasegawa Y. Tropicibacter phthalicus sp. nov., A Phthalate-Degrading Bacterium from Seawater. *Curr Microbiol* 2012;**64**:392–6.

Jeon W, Priscilla L, Park G *et al.* Complete genome sequence of the sulfur-oxidizing chemolithoautotrophic Sulfurovum lithotrophicum 42BKTT. *Stand Genomic Sci* 2017;**12**:54.

Kersh GJ, Lambourn DM, Raverty SA *et al.* Coxiella burnetii Infection of Marine Mammals in the Pacific Northwest, 1997–2010. *J Wildl Dis* 2012;**48**:201–6.

Kitamura R, Miura N, Ito M *et al.* Specific Detection of Coral-Associated *Ruegeria*, a Potential Probiotic Bacterium, in Corals and Subtropical Seawater. *Mar Biotechnol* 2021;**23**:576–89.

Kleindienst S, Herbst F-A, Stagars M *et al.* Diverse sulfate-reducing bacteria of the *Desulfosarcina/Desulfococcus* clade are the key alkane degraders at marine seeps. *ISME J* 2014;**8**:2029–44.

Kopprio GA, Cuong LH, Luyen ND *et al.* Carrageenophyte-attached and planktonic bacterial communities in two distinct bays of Vietnam: Eutrophication indicators and insights on ice-ice disease. *Ecol Indic* 2021;**121**:107067.

Kurilenko V V, Christen R, Zhukova N V *et al.* Granulosicoccus coccoides sp. nov., isolated from leaves of seagrass (*Zostera marina*). *Int J Syst Evol Microbiol* 2010;**60**:972–6.

Laas P, Ugarelli K, Travieso R *et al.* Water Column Microbial Communities Vary along Salinity Gradients in the Florida Coastal Everglades Wetlands. *Microorg*  2022;**10**, DOI: 10.3390/microorganisms10020215.

Lamb JB, van de Water JAJM, Bourne DG *et al.* Seagrass ecosystems reduce exposure to bacterial pathogens of humans, fishes, and invertebrates. *Science (80- )* 2017;**355**:731–3.

Lécuyer H, Audibert J, Bobigny A *et al.* Dolosigranulum pigrum Causing Nosocomial Pneumonia and Septicemia. *J Clin Microbiol* 2007;**45**:3474–5.

Li L, Peng C, Yang Z *et al.* Microbial communities in swamps of four mangrove reserves driven by interactions between physicochemical properties and microbe in the North Beibu Gulf, China. *Environ Sci Pollut Res* 2022;**29**:37582–97.

Li P, Wu S, Yin H *et al.* Bacterial community diversity and dynamics of Dongzhai harbor mangrove soils in China. *Can J Microbiol* 2019;**65**:703–12.

Lim GE, Haygood MG. “*Candidatus* Endobugula glebosa,” a Specific Bacterial Symbiont of the Marine Bryozoan *Bugula simplex*. *Appl Environ Microbiol* 2004;**70**:4921–9.

Lopanik N, Lindquist N, Targett N. Potent cytotoxins produced by a microbial symbiont protect host larvae from predation. *Oecologia* 2004;**139**:131–9.

Martin BC, Alarcon MS, Gleeson D *et al.* Root microbiomes as indicators of seagrass health. *FEMS Microbiol Ecol* 2020;**96**, DOI: 10.1093/femsec/fiz201.

Martin BC, Middleton JA, Skrzypek G *et al.* Composition of Seagrass Root Associated Bacterial Communities Are Linked to Nutrients and Heavy Metal Concentrations in an Anthropogenically Influenced Estuary . *Front Mar Sci*  2022;**8**.

Miroshnichenko ML, Kostrikina NA, Chernyh NA *et al.* *Caldithrix abyssi* gen. nov., sp. nov., a nitrate-reducing, thermophilic, anaerobic bacterium isolated from a Mid-Atlantic Ridge hydrothermal vent, represents a novel bacterial lineage. *Int J Syst Evol Microbiol* 2003;**53**:323–9.

Mishra AK, Mohanraju R. Epiphytic Bacterial Communities in Seagrass Meadows of Oligotrophic Waters of Andaman Sea. *Open Access Libr J* 2018;**5**, DOI: https://doi.org/10.4236/oalib.1104388.

Mußmann M, Ishii K, Rabus R *et al.* Diversity and vertical distribution of cultured and uncultured Deltaproteobacteria in an intertidal mud flat of the Wadden Sea. *Environ Microbiol* 2005;**7**:405–18.

Orsi WD, Smith JM, Liu S *et al.* Diverse, uncultivated bacteria and archaea underlying the cycling of dissolved protein in the ocean. *ISME J* 2016;**10**:2158–73.

Petersen JM, Kemper A, Gruber-Vodicka H *et al.* Chemosynthetic symbionts of marine invertebrate animals are capable of nitrogen fixation. *Nat Microbiol* 2016;**2**:16195.

Quinn RA, Metzler A, Smolowitz RM *et al.* Exposures of *Homarus americanus* Shell to Three Bacteria Isolated from Naturally Occurring Epizootic Shell Disease Lesions. *J Shellfish Res* 2012;**31**:485–93.

Raj Sharma A, Zhou T, Harunari E *et al.* Labrenzbactin from a coral-associated bacterium *Labrenzia* sp. *J Antibiot (Tokyo)* 2019;**72**:634–9.

Reusch TBH, Schubert PR, Marten S-M *et al.* Lower *Vibrio* spp. abundances in *Zostera marina* leaf canopies suggest a novel ecosystem function for temperate seagrass beds. *Mar Biol* 2021;**168**:149.

Riclea R, Gleitzmann J, Bruns H *et al.* Algicidal lactones from the marine *Roseobacter* clade bacterium *Ruegeria pomeroyi*. *Beilstein J Org Chem* 2012;**8**:941–50.

Rinke C, Schmitz-Esser S, Loy A *et al.* High genetic similarity between two geographically distinct strains of the sulfur-oxidizing symbiont ‘Candidatus Thiobios zoothamnicoli.’ *FEMS Microbiol Ecol* 2009;**67**:229–41.

Rosales SM, Clark AS, Huebner LK *et al.* *Rhodobacterales* and *Rhizobiales* Are Associated With Stony Coral Tissue Loss Disease and Its Suspected Sources of Transmission. *Front Microbiol* 2020;**11**.

Shah V, Chang BX, Morris RM. Cultivation of a chemoautotroph from the SUP05 clade of marine bacteria that produces nitrite and consumes ammonium. *ISME J* 2017;**11**:263–71.

Shin N-R, Roh SW, Kim M-S *et al.* *Tropicimonas sediminicola* sp. nov., isolated from marine sediment. *Int J Syst Evol Microbiol* 2012;**62**:2424–9.

Tomáš G, Nicola B, Jan M *et al.* Cyanochelins, an Overlooked Class of Widely Distributed Cyanobacterial Siderophores, Discovered by Silent Gene Cluster Awakening. *Appl Environ Microbiol* 2022;**87**:e03128-20.

Ureta A, Alvarez B, Ramón A *et al.* Indentification of *Acetobacter diazotrophicus*, *Herbaspirillum seropedicae* and *Herbaspirillum rubrisubalbicans* using biochemical and genetic criteria. *Plant Soil* 1995;**172**:271–7.

Wang X, Yu M, Wang L *et al.* Comparative genomic and metabolic analysis of manganese-oxidizing mechanisms in *Celeribacter manganoxidans* DY25T: Its adaptation to the environment of polymetallic nodules. *Genomics* 2020;**112**:2080–91.

Weidner S, Arnold W, Stackebrandt E *et al.* Phylogenetic Analysis of Bacterial Communities Associated with Leaves of the Seagrass *Halophila stipulacea* by a Culture-Independent Small-Subunit rRNA Gene Approach. *Microb Ecol* 2000;**39**:22–31.

Zaynab M, Chen H, Chen Y *et al.* Signs of biofilm formation in the genome of *Labrenzia* sp. PO1. *Saudi J Biol Sci* 2021;**28**:1900–12.

Zhang X, Liu S, Jiang Z *et al.* Gradient of microbial communities around seagrass roots was mediated by sediment grain size. *Ecosphere* 2022;**13**:e3942.

Zhang Y, Ma Y, Zhang R *et al.* Metagenomic Resolution of Functional Diversity in Copper Surface-Associated Marine Biofilms . *Front Microbiol*  2019;**10**.
